# Prolyl isomerization of FAAP20 catalyzed by PIN1 regulates the Fanconi anemia pathway

**DOI:** 10.1371/journal.pgen.1007983

**Published:** 2019-02-21

**Authors:** Jingming Wang, Bryan Chan, Michael Tong, YiTing Paung, Ukhyun Jo, Dwight Martin, Markus Seeliger, John Haley, Hyungjin Kim

**Affiliations:** 1 Department of Pharmacological Sciences, Stony Brook University, Stony Brook, New York, United States of America; 2 Department of Chemistry, Stony Brook University, Stony Brook, New York, United States of America; 3 Department of Pathology, Proteomics Center, Stony Brook University, Stony Brook, New York, United States of America; 4 Stony Brook Cancer Center, Stony Brook University School of Medicine, Stony Brook, New York, United States of America; Stanford University School of Medicine, UNITED STATES

## Abstract

The Fanconi Anemia (FA) pathway is a multi-step DNA repair process at stalled replication forks in response to DNA interstrand cross-links (ICLs). Pathological mutation of key FA genes leads to the inherited disorder FA, characterized by progressive bone marrow failure and cancer predisposition. The study of FA is of great importance not only to children suffering from FA but also as a model to study cancer pathogenesis in light of genome instability among the general population. FANCD2 monoubiquitination by the FA core complex is an essential gateway that connects upstream DNA damage signaling to enzymatic steps of repair. FAAP20 is a key component of the FA core complex, and regulated proteolysis of FAAP20 mediated by the ubiquitin E3 ligase SCF^FBW7^ is critical for maintaining the integrity of the FA complex and FA pathway signaling. However, upstream regulatory mechanisms that govern this signaling remain unclear. Here, we show that PIN1, a phosphorylation-specific prolyl isomerase, regulates the integrity of the FA core complex, thus FA pathway activation. We demonstrate that PIN1 catalyzes *cis-trans* isomerization of the FAAP20 pSer48-Pro49 motif and promotes FAAP20 stability. Mechanistically, PIN1-induced conformational change of FAAP20 enhances its interaction with the PP2A phosphatase to counteract SCF^FBW7^-dependent proteolytic signaling at the phosphorylated degron motif. Accordingly, PIN1 deficiency impairs FANCD2 activation and the DNA ICL repair process. Together, our study establishes PIN1-dependent prolyl isomerization as a new regulator of the FA pathway and genomic integrity.

## Author summary

Fanconi anemia (FA) is a devastating disease of children that leads to birth defects, bone marrow failure, and a variety of cancers early in their lives. Germ-line mutations in FA genes disrupt the DNA repair process, namely the FA pathway, resulting in genome instability and clinical features of FA patients. Thus, understanding the molecular mechanisms by which the FA pathway is regulated is critical for alleviating the burden of children suffering from FA and related cancer. A critical step in this pathway is the monoubiquitination of FANCD2 by a multi-subunit ubiquitin E3 ligase called the FA core complex, and the FAAP20 subunit is required for its functional integrity. Here, we show that proline-directed structural change of FAAP20 catalyzed by the PIN1 prolyl *cis-trans* isomerase is essential for the FAAP20 stability by counteracting phosphorylation-dependent proteolytic signaling of FAAP20 and thus promotes FANCD2 activation and DNA repair. Our findings reveal how PIN1-mediated phosphorylation signaling cascade and proteolysis preserves genomic integrity and how its deregulation is associated the pathogenesis of FA. Our knowledge on a new regulatory mechanism governing FA pathway activation may lead to the development of a new target for FA and FA-related malignancy.

## Introduction

The Fanconi anemia (FA) DNA repair pathway resolves DNA interstrand cross-links (ICLs) and other obstacles encountered during DNA replication [1,2]. Germ-line mutation of at least 22 genes involved in this pathway not only causes a childhood blood disorder of bone marrow failure, FA, but also predisposes affected children to a variety of cancers, highlighting the role of the FA pathway as a tumor suppressor mechanism that preserves genomic integrity [3,4]. Central to this pathway is FANCD2 activation, triggered by its monoubiquitination via a multi-subunit ubiquitin E3 ligase, the FA core complex, which targets the FANCD2-FANCI heterodimeric complex to DNA lesions to recruit structure-specific nucleases and initiate nucleolytic incision of cross-linked DNA [5,6]. Thus, FANCD2 monoubiquitination by the FA core complex constitutes an essential gateway to connect the DNA damage response (DDR) to enzymatic steps of DNA ICL repair. Monoubiquitinated FANCD2 is also required for maintaining DNA replication fork integrity independently of DNA ICL processing [7,8]. The FA core complex consists of at least eight FA gene products associated with several accessory proteins and exhibits modular features to promote the activity of the catalytic E3 ligase core [9–11]. Each subunit is under the control of numerous posttranslational modifications, implicating multiple layers of regulation in response to DNA damage and replication checkpoint [12]. The FANCA-FANCG-FAAP20 subcomplex constitutes a structural module to maintain the integrity of the FA complex and supports its localization to the sites of DNA lesions [10,11]. The 20 kD FA-associated protein FAAP20 directly interacts with FANCA and promotes its stability [13–15]. In the absence of FAAP20, the degron motif of FANCA is exposed to undergo SUMO-dependent proteolytic degradation, leading to the loss of the FA core complex integrity and thus a defect in FANCD2 monoubiquitination [16]. Accordingly, a patient-derived mutation that disrupts the FANCA-FAAP20 interaction causes FA-like phenotypes [16]. The dynamics of the FANCA-FAAP20 interaction is regulated by FAAP20 degradation, which is mediated by the SKP1-CUL1-F-Box/FBW7 (SCF^FBW7^) ubiquitin E3 ligase complex [17]. Specifically, glycogen synthase kinase 3β (GSK3β)-dependent phosphorylation of FAAP20 at the Cdc4 phospho-degron (CPD) motif is recognized by FBW7 to trigger polyubiquitination and proteasome-dependent FAAP20 degradation [17]. Hence, phosphorylation-dependent ubiquitin signaling plays an essential role in regulating the FANCA-FAAP20 interaction and FA pathway activation. Nevertheless, the upstream signaling that governs FAAP20 phosphorylation status and its detailed mode of action for FAAP20 degradation remain uncharacterized.

The reversible phosphorylation-dependent ubiquitin-proteasome system (UPS) is a fundamental regulatory mechanism for protein degradation. As exemplified by FBW7-dependent FAAP20 degradation, phosphorylation of the phospho-degron motif allows proteins to be recognized by a ubiquitin E3 ligase and delivered to the proteasome. Meanwhile, given the rapid phosphorylation-dephosphorylation event mediated by kinases and phosphatases, catalysis by peptidyl-prolyl *cis-trans* isomerase NIMA-interacting 1 (PIN1) is often considered a key rate-determining step in controlling phosphorylation-dependent signaling [18]. PIN1 specifically recognizes a phosphorylated Ser or Thr residue preceding a Pro (pSer/Thr-Pro). By catalyzing the Pro *cis-trans* isomerization that converts substrates into a conformation that is favorable or refractory to downstream signaling, PIN1 acts as a molecular switch to control diverse cellular functions, including proteolysis [19]. Accordingly, previous studies have established the role of PIN1 in regulating the stability of oncoproteins and tumor suppressors in multiple cellular processes, including transcriptional regulation (c-Jun, c-Myc, p53), cell cycle (Cyclin D1 & E), and cell death (MCL-1) [20–27]. Notably, many of these substrates are also substrates for SCF^FBW7^, implying a complex interplay at the PIN1-SCF^FBW7^ phospho-dependent ubiquitin signaling axis to modulate substrate ubiquitination and degradation. Interestingly, a recent study has revealed the role of PIN1 in promoting degradation of CtIP, a mediator of double-strand break (DSB) repair, thereby connecting PIN1 signaling to DNA repair processes [28]. However, the molecular details of how PIN1 regulates its substrates associated with DNA repair are only beginning to be understood.

Here, we identify PIN1 as a new regulator of the FA pathway. We provide evidence that FAAP20 is a new substrate of PIN1 and that PIN1 antagonizes proteolytic signaling of FAAP20 degradation mediated by SCF^FBW7^, thus promoting the integrity of the FA core complex and FANCD2 activation. Together, our study uncovers a new role for the prolyl isomerase PIN1 in governing the DNA ICL repair process and genomic integrity. Given that PIN1 is deregulated in many human cancers, our findings also provide insights into how the disruption of FA pathway signaling may be connected to the genome instability of PIN1-related cancers.

## Results

### The FANCA binding-defective FAAP20 mutant is prone to prolyl isomerization

**Fig 1:**
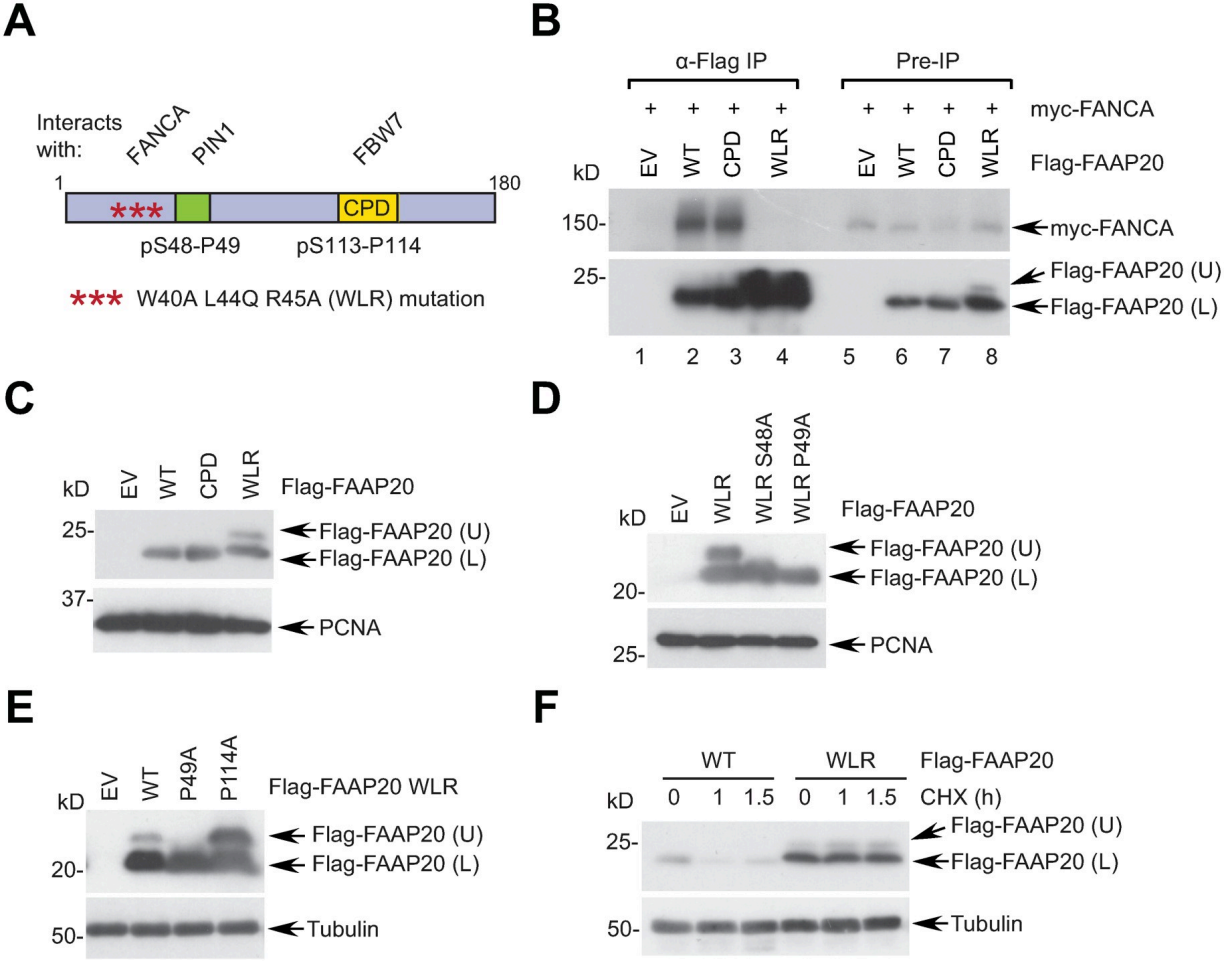
The FANCA binding-defective FAAP20 mutant is isomerized at the pS48-P49 motif. **(A)** A schematic of the FAAP20 structure, displaying the pS48-P49 motif for PIN1 binding and the pS113-P114 CPD phospho-degron motif for FBW7 binding. The N-terminal region of FAAP20 mediates its interaction with FANCA, which is disrupted by W40A, L44Q, and R45A (WLR) mutations. **(B)** 293T cells transiently transfected with indicated plasmids were subjected to anti-Flag immunoprecipitation (IP) and Western blotting (WB). CPD: S113A & S117A mutation. The slower migrating Flag-FAAP20 isoform is designated as (U: upper) in contrast to the faster migrating isoform (L: lower). WB analysis of U2OS cells expressing Flag-FAAP20 wild-type (WT) or various mutants. EV: empty vector. **(C-E)** 293T cells transiently transfected with indicated plasmids were analyzed by WB against anti-Flag and anti-PCNA antibodies. **(F)** U2OS cells transiently expressing Flag-FAAP20 WT or WLR mutant were treated with 50 μg/mL cycloheximide (CHX) for the indicated times, and FAAP20 degradation was monitored by WB.

FANCA and FAAP20 interact and stabilize each other in the FA core complex [13,15,16]. We and others have previously shown that the N-terminal region of FAAP20 is required for the FANCA interaction (Fig 1A) [13,14]. While further charactering the FAAP20-FANCA interaction via extensive mutagenesis, we serendipitously found that one FAAP20 mutant (W40A, L44Q, R45A; hereinafter WLR), which fails to interact with FANCA, exhibits an additional, slower-migrating upper isoform during SDS-PAGE (Fig 1B; lane 8). This additional band was specific to the WLR mutant, and not seen with the CPD degron mutant (Fig 1C). This drastic mobility shift may reflect a conformational change of the proline backbone, which often persists even under denaturing conditions, as previously seen in the PIN1 substrate ATR [29]. Notably, mass spectrometry analysis of both Flag-FAAP20 WLR isoforms revealed that the upper form is phosphorylated at Ser48 adjacent to Pro49 (S1A–1C Fig). Since the phosphorylated Ser-Pro motif is known to be a target for PIN1-catalyzed *cis-trans* isomerization, we determined whether pS48-P49 is responsible for the structural change of the FAAP20 WLR mutant. Indeed, mutations either in the phosphorylated residue Ser48 or in the isomerizing residue Pro49 abolished the upper form (Fig 1D). Overexpression of PIN1 increased the ratio of the upper versus lower form as well as overall FAAP20 WLR levels, while PIN1 knockdown decreased the levels of the upper form (S1D Fig). FAAP20 contains two pSer-Pro motifs, one of which we previously defined as a degron motif that is recognized by FBW7 [17]. Some of the CPD of FBW7 substrates have previously been shown to undergo isomerization by PIN1, thereby directly affecting signaling centered on the CPD [22]. Nevertheless, unlike the Pro49 mutation, disruption of the Pro at the CPD did not abolish the upper form of the FAAP20 WLR (Fig 1E). Interestingly, the FAAP20 WLR mutant was much more stable than wild-type (WT) upon inhibition of nascent protein synthesis by cycloheximide, despite its inability to interact with its protective partner FANCA, suggesting that downstream proteolytic signaling is impaired (Fig 1F). Together, these data indicate that the FAAP20 WLR mutation leads to a structural change specific to the pS48-P49 motif and influences FAAP20 stability.

### FAAP20 interacts with PIN1 in a phosphorylation-dependent manner

**Fig 2:**
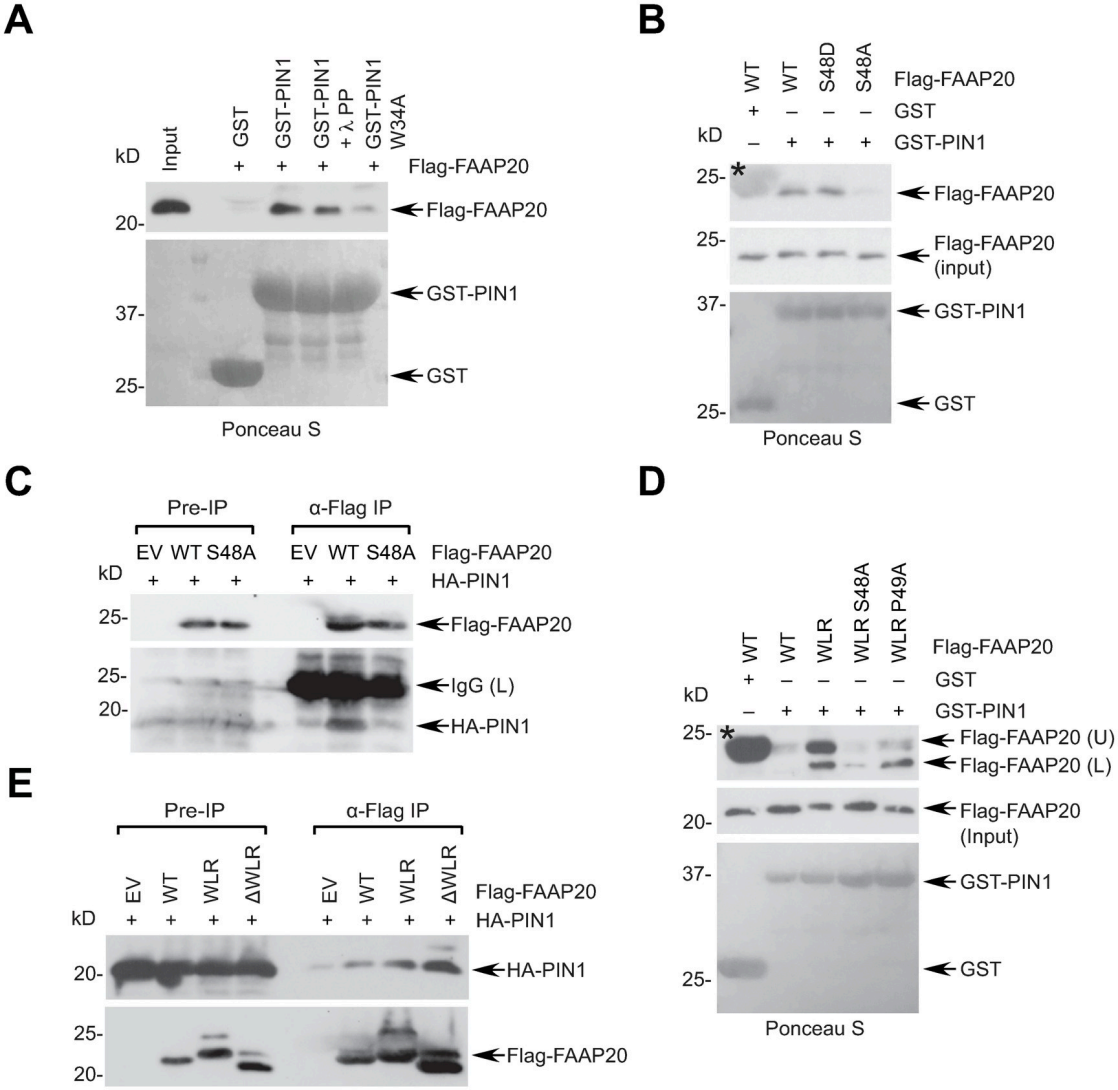
FAAP20 interacts with PIN1 in a phosphorylation-dependent manner. **(A**, **B)** In vitro transcribed and translated (IVTT) FAAP20 WT or mutants were incubated with glutathione beads bound with GST- or GST-PIN1 WT or W34A substrate-binding mutant and analyzed by WB. The input for recombinant GST proteins was analyzed by Ponceau S staining. Where indicated, 20 U/μL lambda protein phosphatase (λ PP) was incubated at 30 °C for 30 min. * denotes GST signal visualized nonspecifically during anti-Flag immunoblots. **(C)** 293T cells transfected with indicated plasmids were subjected to anti-Flag IP and WB. **(D)** GST pull-down of IVTT FAAP20 WT or WLR mutant variants. Note the appearance of the Flag-FAAP20 (U) isoform upon being pulled down by GST-PIN1. (**E**) Enhanced interaction between HA-PIN1 and Flag-FAAP20 WLR (W40, L44Q, R45A) or ΔWLR (a.a.40-45 deletion) revealed by anti-Flag IP and WB of 293T cell lysates.

Our data from the FAAP20 WLR mutant raises the possibility that the pS48-P49 motif of WT FAAP20 could be a physiological target of PIN1. Thus, we determined whether FAAP20 interacts with PIN1 through the pS48-P49 motif. To this end, we purified GST-tagged PIN1 from *E*. *coli* and incubated with in vitro transcribed and translated (IVTT) Flag-tagged FAAP20. GST-PIN1 directly interacted with Flag-FAAP20 WT, whereas the PIN1 W34A substrate-binding mutant [30] failed to do so (Fig 2A). GST-PIN1 also pulled down endogenous FAAP20 from cell lysates (S2A Fig). Similarly, treatment with lambda protein phosphatase also decreased the interaction, arguing for the requirement of FAAP20 phosphorylation for the PIN1 interaction (Fig 2A). Indeed, the non-phosphorylatable FAAP20 S48A mutant was unable to interact with PIN1 in vitro, whereas the WT or the phospho-mimic S48D mutant retained the interaction (Fig 2B). Moreover, Flag-FAAP20 WT, but not S48A, immunoprecipitated HA-PIN1 from cell lysates (Fig 2C). Notably, GST-PIN1 interacted with the WLR mutant stronger than with WT FAAP20 in vitro and induced the formation of the upper form while pulling down the FAAP20 WLR, indicating that enhanced PIN1 interaction renders the FAAP20 WLR more susceptible to the action of PIN1 and subsequent isomerization (Fig 2D). In contrast, the IVTT FAAP20 WLR itself did not exhibit its shifted isoform when it was immunoprecipitated alone in vitro in the absence of PIN1, indicating that the isoform directly results from the structural change induced by PIN1, rather than representing a posttranslational modification that may have occurred during incubation (S2B Fig). We also showed that the FAAP20 WLR point or deletion mutants interact more strongly with PIN1 in the cells in comparison to WT, suggesting that increased affinity of the FAAP20 WLR mutant to PIN1, perhaps due to the change of conformation near the pS48-P49 motif caused by the disruption of the adjacent WLR region, allows enhanced isomerization and appearance of two isoforms (Fig 2E).

### PIN1 catalyzes isomerization of the phosphorylated FAAP20 S48-P49 motif

**Fig 3:**
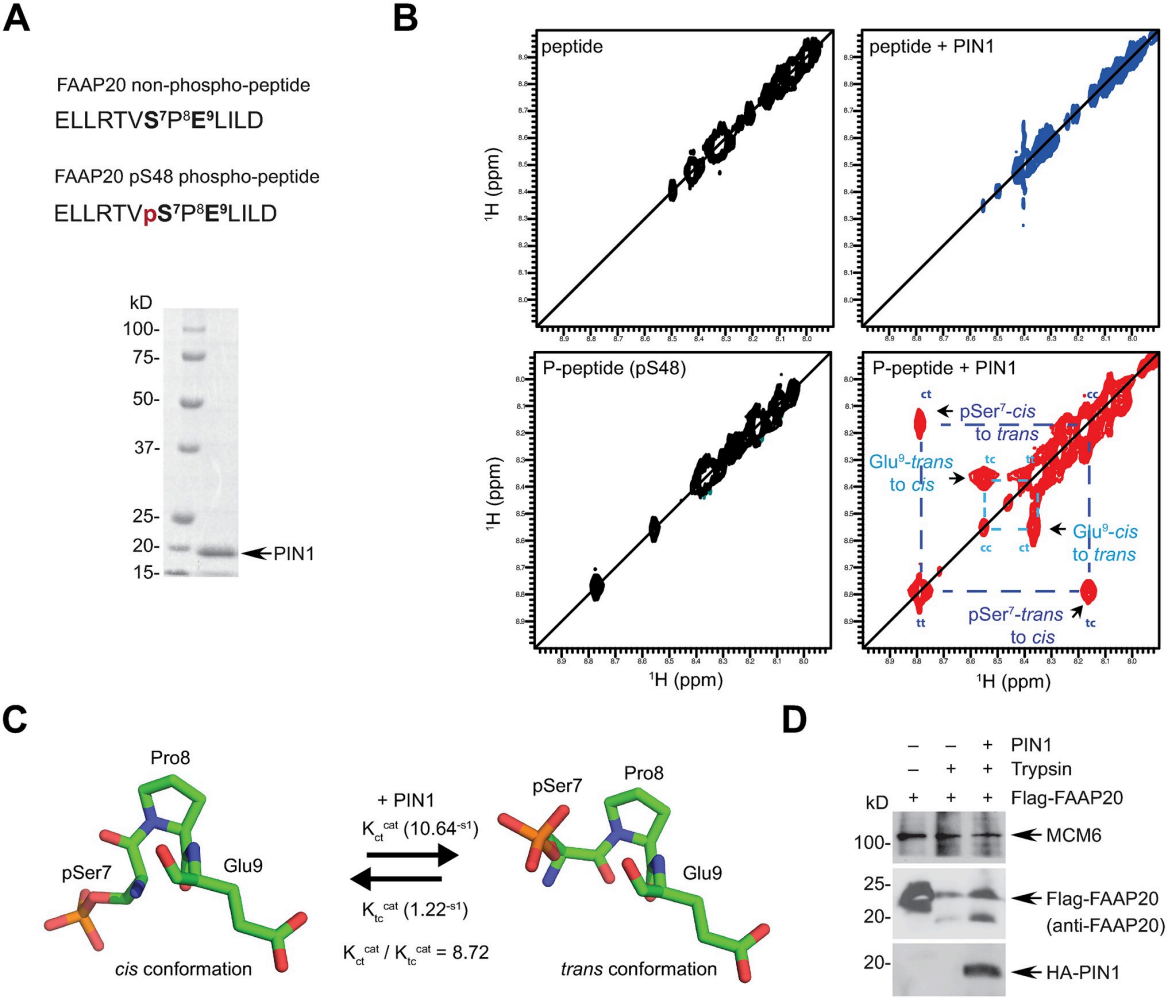
PIN1 catalyzes the isomerization of FAAP20 and induces its conformational change. **(A)** (Top) The FAAP20 peptides used in NMR study. pSer7 and Glu9, which precedes and follows Pro8, respectively, correspond to the pSer48-Pro49-Glu50 amino acid residues of FAAP20. (Bottom) coomassie blue staining of purified recombinant His-tagged PIN1. **(B)** 2D ROESY spectra of 2 mM phospho-peptide alone (bottom left) and peptide mixed with 0.03 mM His-PIN1 (bottom right) for 90 ms. Arrows denote cross-peaks representing *cis-trans* conformational exchange of pSer7 (blue) and Glu9 (pale blue). Dotted lines indicate the amide protons of pSer7 and Glu8 that correlate to the cross-peaks. As a control, a non-phosphorylated peptide was incubated with PIN1 for 300 ms (top left and right). **(C)** Illustration of the conformational exchange of the pFAAP20 peptide by PIN1. The isomerization rates of pSer7 reveal an enhanced *cis-trans* conformational exchange rate by 8.72-fold. **(D)** Where indicated, IVTT Flag-FAAP20 was pre-incubated with 10 ng/μL His-PIN1 followed by 500 ng/mL trypsin at 30 °C for 1 min to undergo limited proteolytic digestion, and the degradation pattern was visualized by anti-FAAP20 WB.

To further support the idea that FAAP20 is a substrate of PIN1, we monitored the conformational change of FAAP20 catalyzed by PIN1 using NMR spectroscopy. To this end, we synthesized FAAP20 peptides either non-phosphorylated or phosphorylated at Ser48 (Fig 3A). Each peptide was incubated with PIN1, and the *cis-trans* conformational exchange of the pSer and Glu residues flanking the Pro residue in the peptide was monitored by ^1^H-^1^H (2D) ROESY (rotating frame Overhause effect spectroscopy) [31]. In this experiment diagonal-peaks corresponding to the amide protons of pSer7 and Glu9 in their *cis* (cc) and *trans* (tt) conformations were studied. Cross-peaks that have the same sign as the diagonal peaks indicate conformational exchange between two distinct conformations, but cross-peaks that have the opposite sign indicate an NOE (Nuclear Overhauser effect). In the absence of PIN1, no exchange cross-peaks were detected, suggesting that conformational exchange between the *cis* and *trans* conformations was too slow to be detected (Fig 3B, bottom left). In contrast, exchange cross-peaks were observed in the phospho-peptide upon PIN1 incubation, indicating evidence of conformational exchange (Fig 3B, bottom right). As a control, incubation of a non-phosphorylated peptide with PIN1 did not generate any notable exchange cross-peaks (Fig 3B, top). These data suggest that substantially greater conformational exchange occurs in the presence of PIN1, and that PIN1 specifically recognizes the phosphorylated S48-P49 motif of FAAP20 to catalyze its isomerization. We also determined both forward (K_ct_^cat^) and reverse (K_tc_^cat^) rate constants for the two-state conformational exchange process by analyzing the ratio of the I_tc_ cross-peak intensity to the I_tt_ diagonal-peak intensity (S3A and S3B Fig). Our analysis indicates that the average value of K_ct_^cat^ is almost 9-fold greater than that of K_tc_^cat^, which is consistent with previous reports showing that the forward rate of *cis*-to-*trans* is greater than the reverse rate of *trans*-to-*cis*, and that the *trans* conformation is predominant over the *cis* conformation (Fig 3C) [32–34].

In addition, we further demonstrated that limited proteolysis of IVTT FAAP20 by trypsin is attenuated when FAAP20 was pre-incubated with recombinant PIN1, indicating that full-length FAAP20 adapts a different conformation upon PIN1-induced isomerization in vitro (Fig 3D). Together, these data suggest that PIN1 catalyzes the isomerization of FAAP20.

### PIN1 activity is required for promoting FAAP20 stability

**Fig 4:**
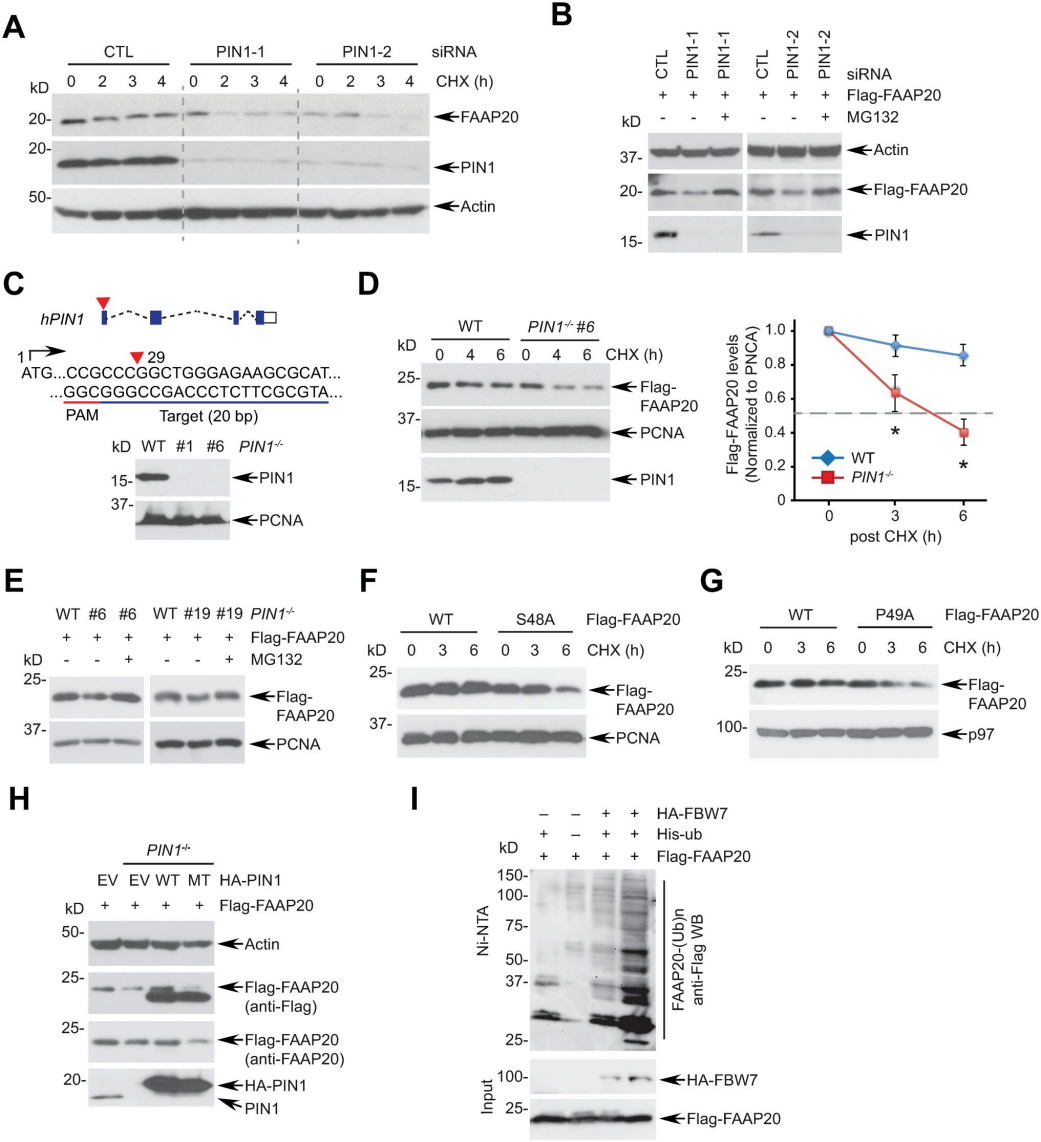
PIN1 is required for maintaining FAAP20 stability. **(A)** U2OS cells transfected with siRNA control (CTL) or PIN1 were treated with 50 μg/mL CHX for the indicated times, and degradation kinetics of endogenous FAAP20 was monitored by WB. **(B)** U2OS cells were serially transfected with indicated siRNA oligos and Flag-FAAP20 encoding plasmid, and cell lysates were analyzed by WB. Where indicated, 10 μM of proteasome inhibitor MG132 was incubated for 6 h before harvest. **(C)** (Top) a schematic for the *PIN1* knockout strategy using CRISPR/Cas9. The 20-nucleotide sgRNA target loci on the bottom strand of exon 1 are marked in blue line along with a PAM sequence in red. The cleavage site for the Cas9 nuclease is indicated by the red triangle, which is 29 nucleotides upstream from the ATG start codon. (Bottom) confirmation of knockout by the WB analysis of U2OS WT (vector-transfected clone) or independent *PIN1*^*-/-*^ clones. **(D)** (Left) U2OS WT or *PIN1*^*-/-*^ #6 clones expressing Flag-FAAP20 were treated with 50 μg/mL CHX for the indicated times, and degradation of Flag-FAAP20 was analyzed by WB. (Right) quantification of Flag-FAAP20 levels by ImageJ. A dotted line indicates the half-life of protein degradation. Data shown are the mean ± SD from two independent experiments. * *P* <0.001 compared to WT, Student’s t-test. **(E)** U2OS WT or *PIN1*^*-/-*^ cells transiently expressing Flag-FAAP20 were left untreated or treated with 10 μM MG132 for 6 h, and cell lysates were analyzed by WB. (**F, G**) U2OS cells expressing Flag-FAAP20 WT or isomerization-defective mutants were treated with 50 μg/mL CHX for the indicated times and analyzed by WB. **(H)** U2OS cells stably expressing PIN1 WT or catalytically dead mutant (K63A/R68A/R69A) were transfected with Flag-FAAP20-encoding plasmid, and cell lysates were analyzed by WB. **(I)** 293T cells transfected with the indicated plasmids were treated with 20 μM MG132 for 4 h, lysed under denaturing conditions, and incubated with Ni-NTA agarose to capture polyubiquitinated Flag-FAAP20. Flag-FAAP20 in Figure 4 was transiently expressed by plasmid transfection.

PIN1-dependent *cis-trans* isomerization often exerts a profound impact on the stability of phosphorylated proteins by affecting the ubiquitin signaling required for proteasomal degradation [19]. Given that we previously identified FAAP20 as a substrate of SCF^FBW7^, which participates in the UPS known to be modulated by PIN1 [30], we determined whether PIN1 affects the stability of FAAP20. Knockdown of PIN1 with two independent siRNAs facilitated the degradation of endogenous FAAP20 upon cycloheximide chase (Fig 4A), and the reduced FAAP20 levels were rescued by proteasome inhibition (Fig 4B), indicating that PIN1 promotes FAAP20 stability in a physiological manner. To further substantiate our findings, we generated *PIN1* knockout (KO) U2OS human osteosarcoma cell lines by CRISPR-Cas9 (Fig 4C). Independent KO clones demonstrated that the half-life of FAAP20 degradation is dramatically shorter in the absence of PIN1 (Fig 4D and S4A Fig). Reduced levels of exogenous FAAP20 expression were rescued by proteasome inhibition, suggesting that PIN1 antagonizes FAAP20 degradation via the proteasome (Fig 4E and S4B Fig). Accordingly, mutations in the Ser48 or Pro49 residues of FAAP20 accelerated FAAP20 degradation, further supporting the idea that FAAP20 isomerization by PIN1 promotes FAAP20 stability (Fig 4F and 4G). The residues in the catalytic PPIase domain of PIN1, including Lys63, Arg68, and Arg69, form a positively charged phosphate-binding loop to coordinate the pSer/Thr of the substrate [35]. Importantly, reconstitution of the catalytically dead PIN1 mutant into *PIN1*^*-/-*^ cells failed to restore the FAAP20 levels reduced by PIN1 deletion, indicating that PIN1 activity is required for stabilizing cellular FAAP20 levels (Fig 4H and S4C Fig). Moreover, Ni-NTA pull-down of ubiquitinated proteins demonstrated that the polyubiquitination of FAAP20 increases in the FAAP20 P49A mutant and in the absence of PIN1, suggesting that FAAP20 is susceptible to degradation without PIN1-induced isomerization (Fig 4I and S4D Fig). Collectively, these data support the idea that PIN1 is required for maintaining FAAP20 stability.

### Protein phosphatase 2A dephosphorylates FAAP20 at the CPD degron motif

**Fig 5:**
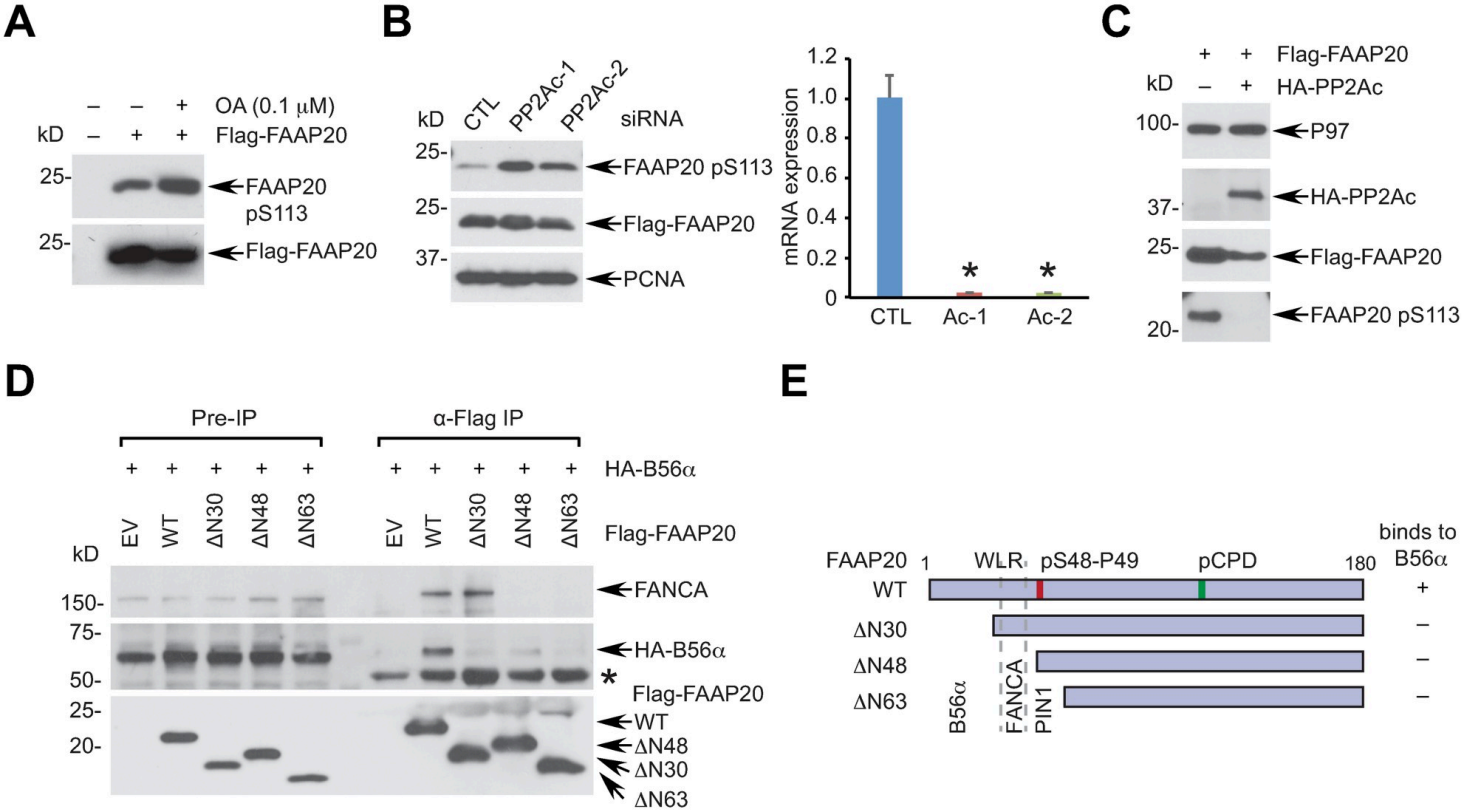
Protein phosphatase 2A dephosphorylates FAAP20 at the CPD motif. **(A)** U2OS cells expressing Flag-FAAP20 were left untreated or treated with 100 nM okadaic acid (OA) for 2 h, and FAAP20 pS113 levels were analyzed by WB using anti-pS113 antibody. **(B)** (Left) U2OS cells were serially transfected with siRNA control or PP2Ac and Flag-FAAP20-encoding plasmid, and FAAP20 pS113 levels were analyzed by WB. (Right) confirmation of PP2Ac knockdown by RT-qPCR. mRNA expression was normalized by GAPDH mRNA (mean ± SD; n = 3 independent experiments). * *P* <0.001, Student’s t-test. **(C)** U2OS cells were transfected with indicated plasmids, and FAAP20 pS113 levels were analyzed by WB. **(D)** 293T cells transfected with HA-B56α and Flag-FAAP20 WT or N-terminal deletion mutants (ΔN30: a.a.1-30 deletion, ΔN48: a.a.1-48 deletion, and ΔN63: a.a.1-63 deletion) were subjected to anti-Flag IP and WB. * IgG heavy chain. **(E)** Summary of the binding regions for B56α, FANCA, and PIN1 revealed in this study.

Phosphorylation at the CPD of FAAP20 is prerequisite for FAAP20 degradation [17]. Thus, we next sought to determine the elements that control the FAAP20 degradation regulated by CPD phosphorylation and PIN1-induced isomerization. Protein phosphorylation is antagonized by phosphatases, and protein phosphatase 2A (PP2A) is a major proline-directed Ser/Thr phosphatase known to regulate diverse cellular processes by counteracting kinase signaling [36]. PP2A has been shown to mediate dephosphorylation of several PIN1 substrates, preferentially recognizing a specific conformation [37]. Hence, we explored the possibility that PP2A is involved in regulating the FAAP20 phosphorylation at the CPD. We had previously generated a FAAP20 antibody that specifically recognizes the pS113 of the CPD, which was used to monitor the CPD phosphorylation status [17] (S5A Fig). Incubation of cells with the PP2A inhibitor okadaic acid (OA) [38] increased the pS113 of FAAP20 (Fig 5A). The PP2A holoenzyme exists as a heterotrimeric complex composed of a catalytic C, a scaffolding A, and a diverse group of regulatory B subunits, which is further classified into four distinct families (B55, B56, B′′, and B′′′), each of which have several isoforms such as α, β, γ, δ, and ε [36]. Specific knockdown of the PP2A catalytic subunit α-isoform (PP2Ac; encoded by *PPP2CA*) with two independent siRNAs also elevated pS113 levels, indicating that the enzymatic activity of PP2A antagonizes FAAP20 phosphorylation at the CPD (Fig 5B and S5B Fig). Conversely, overexpression of PP2A was sufficient to decrease pS113 levels (Fig 5C).

To further explore the direct role of PP2A in FAAP20 dephosphorylation, we determined the interaction of FAAP20 with B56α, the α-isoform of the largest B regulatory subunit family B56 (B′/PR61), which interacts with a substrate and thus confers substrate specificity toward the PP2A holoenzyme [36]. Intriguingly, serial deletion mutagenesis of FAAP20 revealed that the N-terminal amino acid 1–30 residues are required for the interaction with B56α (Fig 5D). As expected, the comparison between the FAAP20 ΔN30 and ΔN48 mutants showed that the amino acid 31–48 residues encompassing the WLR region were necessary for the interaction of FAAP20 with endogenous FANCA. Together, these data suggest that the N-terminal region of FAAP20 is a platform that serially mediates the interactions with multiple regulatory proteins involved in FAAP20 proteolysis, including B56α, FANCA, and PIN1 (Fig 5E). This also indicates that the affinity and activity of PP2A toward FAAP20 may be regulated by the FAAP20 isomerization, which occurs at the adjacent pSer-Pro motif by PIN1.

### PIN1-induced FAAP20 isomerization antagonizes the SCF^FBW7^-dependent ubiquitin signaling at the CPD motif

**Fig 6:**
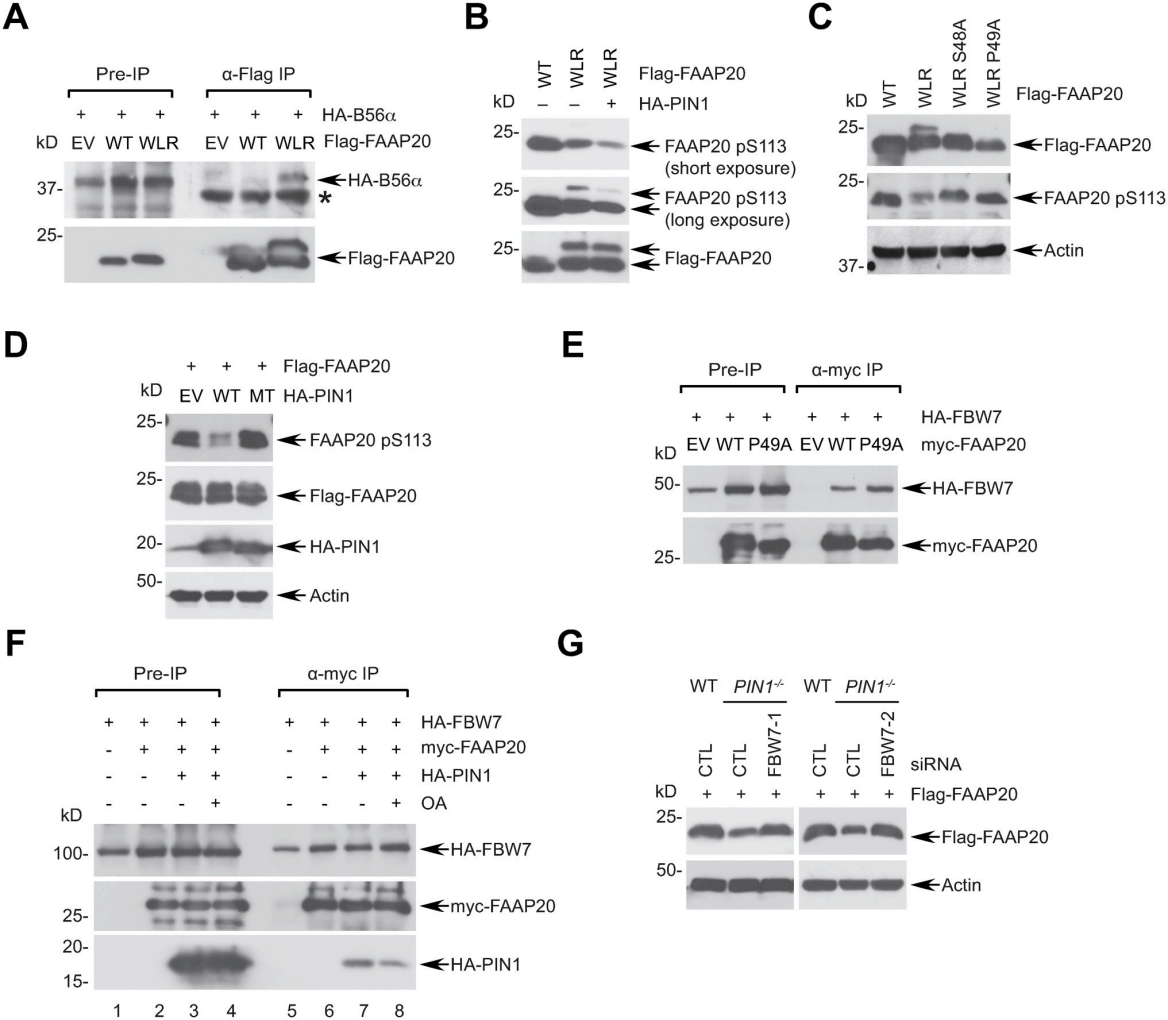
PIN1-induced FAAP20 isomerization promotes PP2A signaling and antagonizes FBW7-dependent FAAP20 degradation. **(A)** 293T cells were transfected with indicated plasmids, and the amount of HA-B56α pulled-down by Flag-FAAP20 was analyzed by anti-Flag IP and WB. **(B, C, D)** U2OS cells were transfected with indicated plasmids, and pS113 levels of Flag-FAAP20 WT or various mutants were analyzed by WB. MT: K63A/R68A/R69A mutant. **(E)** The amount of HA-FBW7 pulled-down by myc-FAAP20 WT or P49A was analyzed by anti-myc IP and WB of 293T cell lysates. **(F)** 293T cells were transfected with indicated plasmids, and the interaction between HA-FBW7 and myc-FAAP20 was analyzed by anti-myc co-IP and WB. (-) denotes empty vector transfection. Where indicated, cells were treated with 50 nM okadaic acid (OA) and 10 μM MG132 for 6 h before harvest. **(G)** U2OS WT or *PIN1*^*-/-*^ cells were serially transfected with two independent siRNA FBW7 oligos (vs. control) and Flag-FAAP20-encoding plasmid, and Flag-FAAP20 levels were analyzed by WB.

Our results thus far raise the possibility that PIN1-mediated FAAP20 isomerization may increase the PP2A holoenzyme association with FAAP20, thereby promoting dephosphorylation of the CPD, which would prevent its interaction with FBW7 and subsequent degradation. Consistent with this idea, we observed that the FAAP20 WLR (i.e. isomerization-prone) mutant binds stronger to B56α in comparison to WT (Fig 6A). On the other hand, the interaction of FAAP20 WT or mutant with GSKβ was largely unaffected, indicating that dephosphorylation may be a rate-limiting step for determining CPD phosphorylation status (S6A Fig). Regarding CPD phosphorylation, the FAAP20 WLR mutant exhibited lower pS113 levels compared to WT, and exogenous expression of PIN1 further decreased pS113 levels (Fig 6B). Importantly, the ratio of pS113 signals in the upper and lower isoforms of FAAP20 was lower than that of the total FAAP20 immunoblot signal, and was further decreased following PIN1 expression, indicating that the isomerized FAAP20 (i.e. upper isoform) is less prone to CPD phosphorylation, and because of its high affinity to PIN1, is more susceptible to dephosphorylation. This idea was further supported by the result showing that CPD phosphorylation was elevated when mutations were introduced to the pS48-p49 motif of the WLR mutant in comparison to the WLR mutation only (Fig 6C). Similarly, exogenous expression of PIN1 WT, but not the catalytically dead mutant, was sufficient to decrease the pS113 levels of WT FAAP20 (Fig 6D). Conversely, the isomerization-defective mutant, FAAP20 P49A, exhibited an increased interaction with FBW7, indicating that the increase in CPD phosphorylation, caused by reduction of PIN1 and PP2A activity, promotes SCF^FBW7^-dependent ubiquitin signaling (Fig 6E). To further support this idea, we examined the interplay among PIN1, PP2A, and FBW7 toward FAAP20 degradation. Co-immunoprecipitation demonstrated that exogenous expression of PIN1 decreased the interaction of FAAP20 with FBW7, which was restored by the inhibition of PP2A activity by OA, indicating that PIN1 counteracts SCF^FBW7^ proteolytic signaling by promoting dephosphorylation of the CPD motif, mediated by PP2A (Fig 6F; compare FBW7 lanes 7 & 8). Accordingly, decreased FAAP20 levels in the absence of PIN1 were rescued by FBW7 depletion, suggesting that PIN1 restricts SCF^FBW7^ activity to promote FAAP20 stabilization (Fig 6G and S6B Fig). Collectively, these data support our model that PIN1-induced conformational change of FAAP20 promotes FAAP20 dephosphorylation at the CPD by PP2A, thereby preventing SCF^FBW7^-dependent FAAP20 degradation.

### PIN1 promotes FANCD2 activation and DNA ICL repair

**Fig 7:**
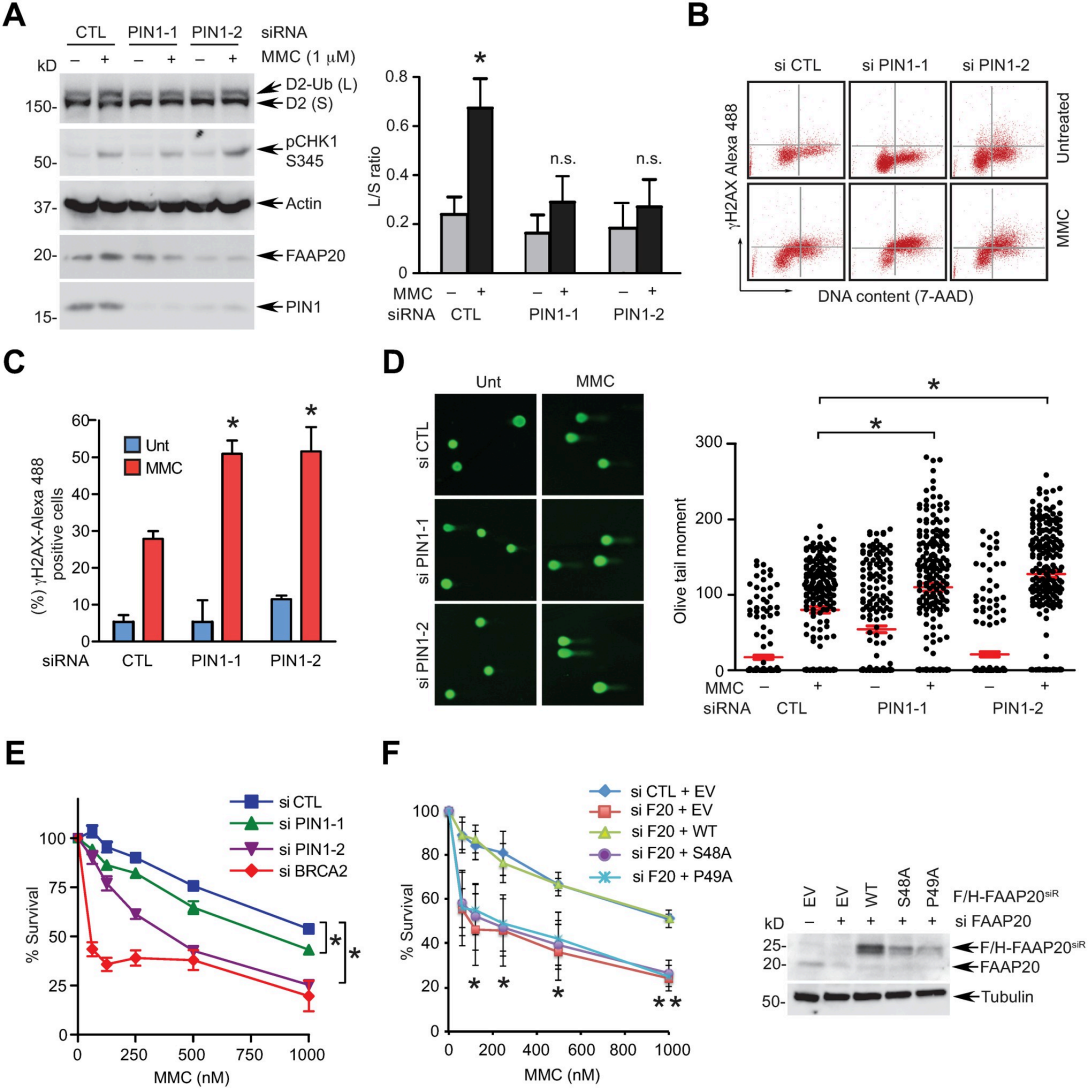
Knockdown of PIN1 impairs the FA pathway. **(A)** U2OS cells transfected with two independent PIN1 siRNAs (vs. control) were treated with 1 μM mitomycin C (MMC) for 8 h, and induction of FANCD2 monoubiquitination (FANCD2-Ub) was analyzed by WB. A representative image is shown, and FANCD2-Ub/FANCD2 (L/S) ratio was quantified by Image J from three independent experiments (right). Mean ± SEM; * *P* <0.05 compared to untreated, two-tailed Student’s t-test. n.s: not significant. **(B)** Representative flow cytometry of Alexa Fluor 488-conjugated γH2AX and 7-AAD staining from siRNA-transfected U2OS cells treated with 50 ng/mL MMC for 18 h. Upper quadrants represent γH2AX-positive cells. **(C)** Quantification of γH2AX-positive cells. Error bar, mean ± SD; n = 3 independent experiments, * *P* <0.01, Student’s t-test. **(D)** (Left) a representative image of comet tails from siRNA-transfected U2OS cells treated with 0.3 μM MMC for 18 h. (Right) plotting of olive tail moment. Mean ± SEM is shown in red. * *P* <0.001, two-tailed Student’s t-test. Representative data from two independent experiments are shown. **(E)** Cell survival assay of U2OS cells transfected with indicated siRNA oligos. Data shown are mean ± SEM from three independent experiments. * *P* <0.05, CTL vs. PIN1 knockdown, paired Student’s t-test. **(F)** (Left) U2OS cells were sequentially transfected with siRNA FAAP20 and siRNA-resistant pMSCV-Flag-HA-FAAP20 WT or indicated mutants, and cellular viability was measured. Data shown are mean ± SD from three independent experiments. * *P* <0.05, WT vs. S48A or P49A reconstitution, ** *P* <0.01, WT vs. S48A or P49A reconstitution, Student’s t-test. (Right) WB analysis of reconstituted FAAP20 WT or mutants in FAAP20-depleted cells.

Disruption of FAAP20 stability impairs the integrity of the FA complex, leading to a defect in FANCD2 activation required for the initiation of DNA ICL repair [13]. Thus, we hypothesized that PIN1 is an unidentified regulatory component of the FA core complex that controls FANCD2 monoubiquitination and examined the role of PIN1 in FA pathway signaling. PIN1 depletion using two independent siRNAs decreased the levels of FANCD2 monoubiquitination induced by a DNA cross-linking agent mitomycin C (MMC), which is visualized by the more slowly migrating, modified FANCD2 (FANCD2-Ub) in an immunoblot, indicating that PIN1 is required for promoting FANCD2 activation (Fig 7A). Under the prolonged treatment of cycloheximide, PIN1 knockdown resulted in accelerated degradation of FANCA, a direct interaction partner of FAAP20 in the FA core complex, which was antagonized by exogenous expression of the FAAP20 CPD mutant that is refractory to degradation by SCF^FBW7^ but proficient for FANCA interaction (S7A Fig). This suggests that defective FA pathway activation in PIN1-deficient cells results primarily from the compromised FA core complex caused by destabilization of FAAP20. Accordingly, cytometry-based quantification of γH2AX, a marker for replication-associated DSBs, revealed that PIN1 knockdown significantly increases the cells with positive γH2AX signals upon MMC treatment when compared to control (Fig 7B and 7C). Furthermore, a comet assay demonstrated that PIN1 depletion increases the levels of DNA breaks upon MMC treatment, together suggesting that defects in the FA pathway caused by PIN1 deficiency results in persistent DNA damage and impaired resolution of DNA lesions (Fig 7D). Accordingly, cells depleted of PIN1 were hypersensitive to MMC, indicating that PIN1 dictates the progression of DNA ICL repair and cellular survival (Fig 7E and S7B Fig). To further substantiate the specific role of FAAP20 isomerization in the FA pathway, we reconstituted siRNA-resistant FAAP20 WT or isomerization-defective mutants in FAA20-depleted cells and examined MMC sensitivity. While FAAP20 WT could complement MMC hypersensitivity of FAAP20-depleted cells, the FAAP20 S48A or P49A mutants failed to do so despite their comparable or higher expression than endogenous FAAP20, indicating that FAAP20 isomerization is required for the function of FAAP20 in the FA pathway (Fig 7F). On the other hand, the WLR mutant, which cannot interact with FANCA despite its increased stability, was not able to complement the FAAP20 deficiency (S7C Fig). The CPD mutant could not fully complement the FAAP20 deficiency either, since FANCA turnover dynamics during DNA ICL repair, which is regulated by FAAP20 phosphorylation and degradation, is also a determinant for DNA ICL repair outcome as previously described [17]. Together, the results of the WLR and CPD mutants further highlight the notion that the effect of PIN1-induced FAAP20 isomerization on DNA ICL repair is largely mediated through the FANCA interaction and the FA core complex.

**Fig 8:**
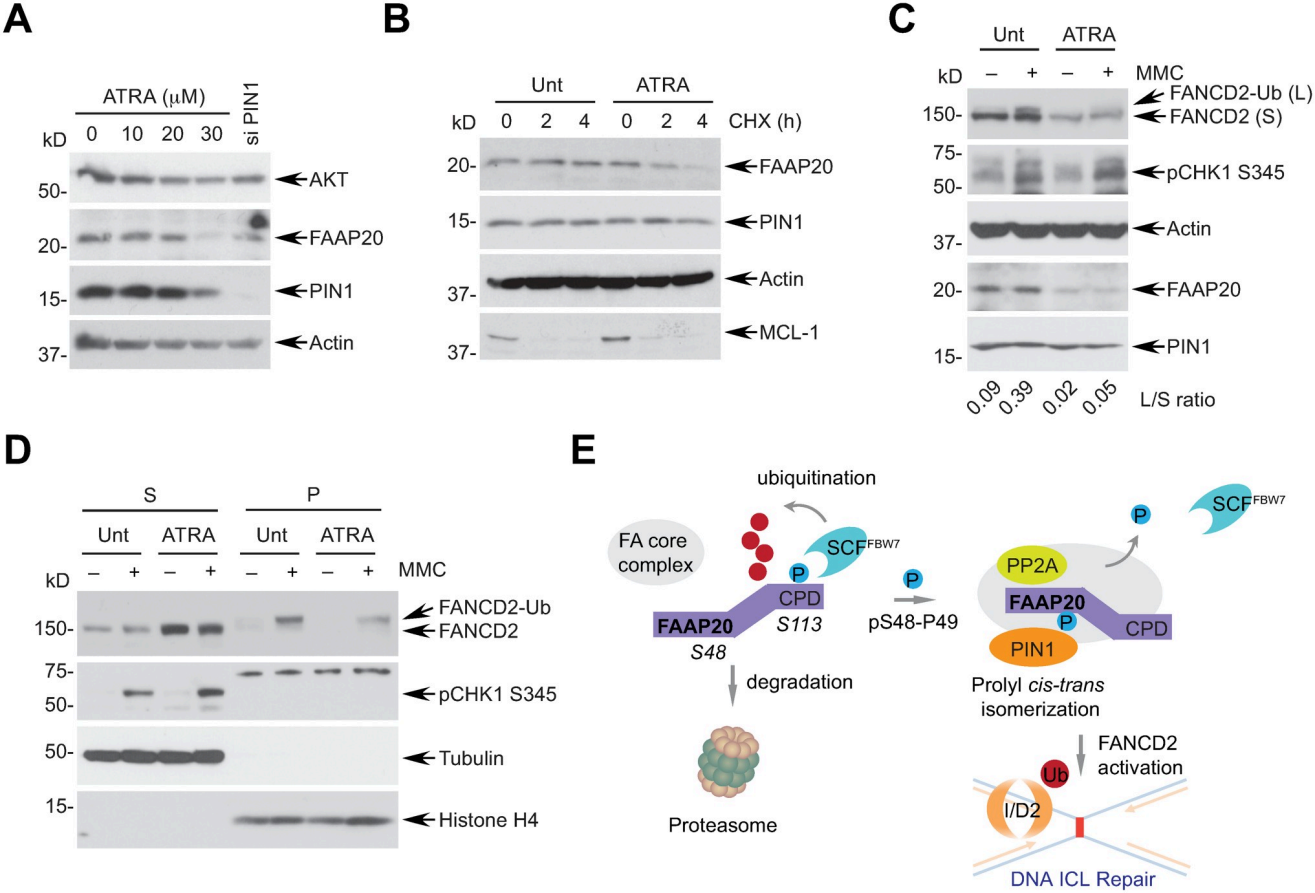
Pharmacological PIN1 inhibition disrupts FANCD2 activation. **(A)** MDA-MB-231 cells were treated with indicated doses of ATRA for 72 h and analyzed by WB with indicated antibodies. As a control, cells were transfected with siRNA PIN1 for 72 h. **(B)** MDA-MB-231 cells treated with 30 μM ATRA for 72 h were treated with 50 μg/mL CHX for the indicated times, and degradation of endogenous FAAP20 was analyzed by WB. **(C)** Cells treated with 30 μM ATRA were challenged with 1 μM MMC for 6 h, and induction of FANCD2 monoubiquitination was analyzed by WB and quantitated by ImageJ. **(D)** ATRA and MMC-treated MDA-MB-231 cells were fractionated into cytosolic/nucleoplasmic (S) and chromatin-enriched (P) fractions, and chromatin association of FANCD2-Ub was analyzed by WB. Localization of tubulin and Histone H4 represents S and P fractions, respectively. **(E)** Model depicting the role of PIN1 in regulating the FA pathway. Phosphorylation of the CPD motif recruits SCF^FBW7^ and subjects FAAP20 for proteasomal degradation. PIN1-induced FAAP20 isomerization at the pS48-P49 motif enhances the interaction of FAAP20 with PP2A to dephosphorylate the CPD motif, thus antagonizing SCF^FBW7^-mediated FAAP20 degradation. Stabilized FAAP20 in the FA core complex promotes FANCD2 activation and DNA ICL repair.

Modulation of FA pathway activity is closely associated with the chemotherapeutic efficacy of DNA cross-linking cytotoxic chemotherapy [39]. Thus, based on our findings, we determined whether pharmacological inhibition of PIN1 is sufficient to disrupt the FA pathway via FAAP20 destabilization in breast cancer, where high levels of PIN1 have been correlated with aggressiveness and chemoresistance [40]. Treatment of MDA-MB-231 triple negative breast cancer (TNBC) cell lines with the recently identified PIN1 inhibitor all-*trans* retinoic acid (ATRA), which binds to the PIN1 active site and degrades PIN1 [41], decreased the levels of FAAP20 in a dose-dependent manner, as well as the levels of AKT, a known target of PIN1, without significantly affecting cellular viability [42] (Fig 8A and S7D Fig). FAAP20 degradation was accelerated in the presence of ATRA, further confirming that PIN1 activity is required for FAAP20 stability (Fig 8B). Accordingly, MDA-MB-231 cells treated with ATRA exhibited less damage-induced FANCD2 monoubiquitination and reduced localization of monoubiquitinated FANCD2 to chromatin, indicating that signaling of FANCD2 activation by the FA core complex is disrupted (Fig 8C and 8D). Collectively, these data allude to the potential of pharmacological PIN1 inhibition as a method for enhancing the chemotherapeutic response of cross-linking regimens by destabilizing FAAP20 and thus disrupting the FA pathway.

## Discussion

### PIN1 as a new regulatory component of the FA pathway

Despite the critical roles of PIN1 in regulating numerous cellular processes, its connection to DNA ICL repair and genome maintenance pathways has remained uncharacterized. Here, we have identified PIN1 as a new regulatory component of the FA core complex in the FA pathway and established the first direct link between PIN1-SCF^FBW7^-mediated proteolysis and DNA ICL repair. Our results propose a model wherein PIN1 maintains the integrity of the FA core complex via phosphorylation-dependent FAAP20 isomerization (Fig 8E). Dissociation of FAAP20 from FANCA in the FA core complex subjects FAAP20 to GSK3β-dependent phosphorylation at the CPD, leading to SCF^FBW7^-dependent polyubiquitination and proteasome delivery. PIN1 antagonizes this process by acting as a molecular switch to catalyze the isomerization of the phosphorylated S48-P49 motif of FAAP20 and induce its conformational change, which enhances its interaction with PP2A, subsequently decreasing CPD phosphorylation and SCF^FBW7^ interaction.

Importantly, our FAAP20 WLR mutant turned out to be a valuable separation-of-function mutant. It has lost its interaction with FANCA and is thus subject to degradation; however, it has also become more susceptible to PIN1-induced isomerization. This unique property allowed us to specifically address the PIN1-PP2A signaling that antagonizes SCF^FBW7^-dependent degradation of FAAP20 when dissociated from FANCA. Since the WLR-deletion mutant still strongly interacts with PIN1 and exhibits two isoforms, we do not believe that the WLR region directly mediates the PIN1 interaction (Fig 2E). Rather, the WLR region may be antagonistic for PIN1 access to the adjacent pS48-P49 motif, and disruption of this region may alter the local conformation of FAAP20, enhancing PIN1 targeting to FAAP20. We propose that the increased FAAP20 stability would allow FAAP20 to favorably associate with the FA core complex, thereby promoting the integrity of the FA core complex and FANCD2 activation upon damage. In other words, cellular levels of FAAP20 that are available to interact with FANCA, even the pool of FAAP20 that may transiently dissociate from FANCA, may be positively maintained by PIN1 in order to sustain FANCA and the FA core complex. PIN1-dependent FAAP20 isomerization may occur during translation before FAAP20 is incorporated into the FA core complex, and PIN1 counteracts the degradation process of FAAP20 to keep adequate levels of FAAP20 available for the interaction with FANCA. Alternatively, PIN1-induced isomerization also antagonizes degradation of FAAP20 while interacting with FANCA by preventing the access of SCF^FBW7^, thus further promoting the stability of the FA core complex. In this regard, PIN1 activity is critical for dictating the outcome of DNA ICL repair processes by modulating integrity of the FA core complex.

It is also tempting to speculate that PIN1-induced FAAP20 isomerization regulates the FAAP20-FANCA interaction dynamics during DNA ICL repair, and thus fine-tunes the activity of the FA core complex. We previously showed that FBW7 depletion alongside prolonged accumulation of FAAP20 impairs DNA ICL repair, indicating that spatiotemporal removal of FANCA-FAAP20, or the FA core complex as a whole, from DNA lesions is critical for the completion of DNA ICL repair [17]. Downregulation of PIN1 activity may trigger degradation of FAAP20, thus facilitating the clearance of the FA core complex to suppress FANCD2 monoubiquitination in the later stage of repair. Indeed, various post-translational modifications of PIN1, including phosphorylation and SUMOylation, affect PIN1 activity, and PIN1 is known to be phosphorylated in a DNA damage-dependent manner [35,43–46]. Thus, elucidating how modification of PIN1 in response to the DNA damage checkpoint regulates PIN1 activity will be an important future direction to more clearly understand how PIN1 contributes to genomic integrity. Interestingly, we observed that the P49A mutation of FAAP20 does not disrupt its interaction with FANCA, indicating that conformational change of FAAP20 by isomerization per se does not directly regulate the FANCA interaction (S7E Fig). In contrast, we consistently notice that the S48A mutation more or less impairs FANCA interaction, indicating that phosphorylation of FAAP20 may regulate its association with the FA core complex independently of isomerization, although its exact role remains to be determined.

### Mechanisms of PIN1 regulation on phosphorylation-dependent ubiquitin signaling

Our study provides important mechanistic insights into how phosphorylation-dependent ubiquitin-proteasome signaling is regulated by PIN1-catalyzed isomerization. Here, we show that PIN1 accelerates conformational changes of phosphorylated FAAP20, which affects its interaction with regulatory proteins, including PP2A and FBW7, thus changing the fate of the protein. This result highlights a complex interplay among prolyl isomerization, phosphorylation, and ubiquitin signaling, which is in agreement with previous studies of known PIN1 substrates. Proline-directed phosphatase PP2A is conformation-specific, and PIN1-induced prolyl isomerization is known to allow PP2A to interact with and dephosphorylate the pSer/Thr-Pro motif of Cdc25C [37]. PIN1 also interacts with the pThr231-Pro motif of tau, which facilitates PP2A-dependent dephosphorylation of hyperphosphorylated tau, restoring its function in microtubule assembly [37,47]. Moreover, PIN1-induced isomerization at the pThr58-Pro motif of c-Myc has been shown to enhance PP2A function to dephosphorylate Ser62, which promotes c-Myc degradation [25]. These studies support the notion that PIN1-induced isomerization is an important regulatory mechanism for controlling PP2A-mediated protein dephosphorylation, which determines the kinetics of substrate degradation and modulates its function. Interestingly, a recent study reported that conformational changes of the epigenetic modulator BRD4 by PIN1 not only prevent its degradation, but also increase its interaction with the downstream transcriptional regulator CDK9 and thus BRD4’s transcriptional activity [48]. By directly visualizing the CPD phosphorylation status that dictates FAAP20 degradation, we were able to provide a mechanism in which a conformational change of FAAP20 by PIN1 modulates the dynamic interaction between PP2A and FBW7 with FAAP20. Currently, it is not clear how a specific conformation of FAAP20 favors the interaction with PP2A or whether PIN1-induced isomerization at the pS48-P49 motif also influences the conformational change at the CPD. Interestingly, we showed that a defined N-terminal region of FAAP20 preceding the pS48-p49 motif is responsible for interacting with the substrate-binding subunit of PP2A and FANCA. This suggests that a local conformational change at the pS48-P49 motif by PIN1 can readily influence the association of FAAP20 with PP2A, although we do not exclude the possibility of FANCA that binds FAAP20 through the WLR region directly affecting the interaction of FAAP20 to PP2A. A detailed structural analysis of this region would be required to reveal how PIN1-induced isomerization affects the dynamic interactions of FAAP20 with its regulatory proteins.

### Exploiting the FA pathway via PIN1 inhibition for cancer therapy

Besides to the pathogenesis of FA, the mechanistic principle developed in our studies has important clinical implications to cancer, since exploiting deregulation of PIN1 activity in the FA pathway could alter the response of cancer cells to cytotoxic chemotherapy or poly ADP-ribose polymerase (PARP) inhibitors [49]. Numerous efforts have been made to develop small molecule inhibitors to modulate reversible monoubiquitination of FANCD2 and inhibit the FA pathway, thereby augmenting the sensitivity of cancer cells to cytotoxic chemotherapy regimens, including platinum [12,50,51]. Intriguingly, PIN1 is widely overexpressed in many human cancers and is associated with poor clinical outcomes [52,53]. In particular, increased activity is often observed in the majority of human breast cancers, and PIN1 is considered to be an essential factor for breast tumorigenesis, as well as cancer stem cells [24,40,54,55]. Accordingly, PIN1 inhibition has been considered as an attractive strategy for cancer therapy [56]. Our data indicate that PIN1 inhibition and subsequent disruption of the FA pathway can potentially function as a chemosensitizer for DNA cross-linking cytotoxic chemotherapy. This may be particularly relevant to the TNBC subtype of breast cancer, which shares similar molecular features to the tumors arising from *BRCA1/FANCS* and *BRCA2/FANCD1*-associated DNA repair deregulation [57]. A recent study also proposed a role of PIN1 in suppressing CtIP, and thus homologous recombination (HR), which may increase error-prone repair and promote tumorigenesis, indicating that PIN1 inhibition could be a general strategy to supplement chemosensitization or exploit the synthetic lethality of PARP inhibition in PIN1-upregulated tumors [28]. Future studies to characterize a comprehensive regulatory network that governs PIN1-PP2A-SCF^FBW7^ signaling will provide important mechanistic insights into the proteolytic control of the FA pathway in preserving genomic integrity and allow for the development of therapeutic strategies to exploit aberrant DNA repair in cancer cells caused by deregulated phosphorylation-dependent ubiquitin signaling.

## Materials and methods

### Cell culture and plasmid construction

U2OS and 293T cell lines were acquired from the American Tissue Culture Collection (ATCC). MDA-MB-231 was a kind gift from Jun Chung (Stony Brook Medicine). Cells were cultured in Dulbecco’s Modified Eagle’s Medium supplemented with 10% fetal bovine serum and 1% penicillin/streptomycin, following standard culture conditions and procedures. *FAAP20*, *FBW7*, and *GSK3β* constructs were previously described [17]. Plasmids encoding GST-PIN1 was a gift from Michael Yaffe (Addgene plasmid #19027), His-PIN1 from Dustin Maly (Addgene plasmid #40773), V245 pCEP-4HA-B56α from David Virshup (Addgene plasmid #14532), and pBABE-zeo PPP2CA from William Hahn (Addgene plasmid #10689). PIN1 cDNA was subcloned into modified pcDNA3-HA or pMSCV-Flag-HA vectors (Invitrogen). Point or deletion mutations were introduced using the QuikChange II XL Site-Directed Mutagenesis (SDM) kit (Agilent Technologies) and confirmed by DNA sequencing (SBU DNA sequencing facility). Stable cell lines were generated by retroviral transduction of pMSCV-Flag-HA-PIN1 constructs using 8 μg/mL polybrene (Sigma-Aldrich), followed by selection with 2 μg/mL puromycin. Viruses were generated from 293T cells that were co-transfected with pMSCV-Flag-HA-PIN1, pCMV-Gag/Pol and pCMV-VSV-G.

### Plasmid and siRNA transfection

Transient plasmid transfection was performed using GeneJuice (Millipore) according to the manufacturer’s protocols. siRNA duplexes were transfected at 25 nM using Lipofectamine RNAiMAX (Invitrogen). The following DNA sequences were targeted by siRNA: Control: 5′-CAGGGTATCGACGATTACAAA-3′; FAAP20: 5′-CACGGTGAGCCCGGAGCTGAT-3′; PIN1-1: 5′-CGGCTACATCCAGAAGATCAA-3′; PIN1-2: 5′-CAGGCCGAGTGTACTACTTCA-3′; FBW7-1: 5′-GTGGAATGCAGAGACTGGAGA-3′; FBW7-2: 5′-CGGGTGAATTTATTCGAAATT-3′; BRCA2: 5′-TTGAAGAATGCAGGTTTAATA-3′ (Qiagen). siRNA sequences for hPP2Ac are 5′-GAACTTGACGATACTCTAAtt-3′ (#1; s10959) and 5′-CCAAACUAUUGUUAUCGUUtt-3′ (#2; s10957) and synthesized from Ambion, Thermo Fisher. Generation of siRNA-resistant FAAP20 was previously described [58].

### Antibodies and chemicals

Antibodies used in this study included: FAAP20 (HPA038829, Sigma-Aldrich), Flag (F1804, Sigma-Aldrich), c-Myc (9E10, Sigma-Aldrich), α-Tubulin (sc-8035, Sigma-Aldrich), FANCD2 (FI-17, Santa Cruz), PCNA (PC-10, Santa Cruz), PIN1 (A302-316A, Bethyl), FANCA (A301-980A, Bethyl), MCL-1 (A302-715A, Bethyl), γ-Tubulin (A302-631A, Bethyl), HA (6E2, Cell Signaling), ubiquitin (P4D1, Cell Signaling), β-Actin (4967, Cell Signaling), p97 (2648, Cell Signaling), pCHK1 S345 (2341, Cell Signaling), γH2AX (2577, Cell Signaling), AKT (9272, Cell Signaling), Histone H4 (07–108, Millipore), BRCA2 (OP95, Millipore), and pS113 FAAP20 (in house; Genscript). Mitomycin C (M5030), Z-Leu-Leu-Leu-al (MG132; C2211), and cycloheximide (C4859), were purchased from Sigma-Aldrich. Okadaic acid (459620) was from EMD Millipore and all-*trans* retinoic acid (ATRA)/Tretinoin (S1653) was from Selleckchem. Drugs were used at the concentrations indicated in the figure legends.

### Western blotting, immunoprecipitation, and subcellular fractionation

Cells were lysed in NETN300 buffer (1% NP40, 300 mM NaCl, 0.1 mM EDTA, and 50 mM Tris [pH 7.5]) supplemented with protease inhibitor cocktail (Roche) and halt phosphatase inhibitor cocktail (Thermo Fisher), resolved by SDS-PAGE, transferred onto PVDF membranes (Millipore), and antibodies were detected using an enhanced chemiluminescence method. Some of immunoblot images were acquired by iBright CL1000 imaging system (Thermo Fisher). For co-immunoprecipitation, 293T cells were lysed in NETN150 buffer (1% NP40, 150 mM NaCl, 0.1 mM EDTA, and 50 mM Tris [pH 7.5]) in the presence of protease and phosphatase inhibitor cocktails and were centrifuged at 15,000 rpm for 10 min at 4 °C. Cell lysates were incubated with anti-Flag M2 affinity gel (A2220, Sigma-Aldrich) or anti-c-Myc agarose affinity gel (A7470, Sigma-Aldrich) for 4 h followed by five washes with NETN150 buffer. Resins were boiled in 2X Laemmli sample buffer and subjected to SDS-PAGE. Subcellular fractionation was performed as previously described [59]. Briefly, cells were lysed using cytoskeleton (CSK) buffer (10 mM Tris [pH 6.8], 100 mM NaCl, 300 mM sucrose, 3 mM MgCl_2_, 1 mM EGTA, 1 mM EDTA, and 0.1% Triton X-100) for 5 min on ice. After centrifugation at 1,500 g for 5 min, the supernatant (S) was separated from the pellet (P), and pellets were sequentially lysed in PBS and 2X boiling lysis buffer (50 mM Tris [pH 6.8], 2% SDS, and 850 mM β-mercaptoethanol).

### Mass spectrometry

After separation via SDS-PAGE and coomassie blue staining, excised gel pieces were destained, reduced, alkylated, and digested with trypsin gold (Promega, V5280), essentially as previously described with minor modifications [60]. The resulting peptide extract was dried and dissolved in a solution of 2% acetonitrile (ACN), 0.1% formic Acid (FA) (buffer A) for analysis by automated microcapillary liquid chromatography-tandem mass spectrometry. Fused-silica capillaries (100 μm inner diameter—i.d.) were pulled using a P-2000 CO_2_ laser puller (Sutter Instruments, Novato, CA) and packed with 10 cm of 5 μm ProntoSil 120-5-C18H (Bischoff Chromatography, Leonberg, Germany) using a pressure bomb. The samples were loaded via an Eksigent NanoLC Autosampler. The column was installed in-line with an Eksigent Nano2D High Performance Liquid Chromatography (HPLC) pump running at 300 nL min^-1^. The peptides were eluted from the column by applying a 115 min gradient from 2% buffer B (98% ACN, 0.1% FA) to 40% buffer B. The gradient was switched from 40% to 80% buffer B over 3 min and held constant for 3 min. Finally, the gradient was changed from 80% buffer B to 2% buffer B over 0.1 min, and then held constant at 2% buffer B for 29 more minutes. The application of a 2.2 kV distal voltage electrosprayed the eluting peptides directly into an LTQ Orbitrap XL ion trap mass spectrometer (Thermo Fisher) equipped with a nano-liquid chromatography electrospray ionization source. Full mass spectra (MS) were recorded on the peptides over a 400 to 2000 *m*/*z* range at 60,000 resolution, followed by top-five MS/MS scans in the ion-trap. Charge state dependent screening was turned on, and peptides with a charge state of +2 or higher were analyzed. Mass spectrometer scan functions and HPLC solvent gradients were controlled by the Xcalibur data system (Thermo Fisher). MS/MS spectra were extracted from the RAW file with ReAdW.exe (http://sourceforge.net/projects/sashimi). The resulting mzXML data files were searched with The GPM X!Tandem and MaXQuant Andromeda search engines against a custom database composed of the Uniprot human proteome with added sequences for common contaminants.

### NMR spectroscopy

The non-phosphorylated and phosphorylated FAAP20 peptides were synthesized from Genscript. All NMR experiments were performed on a Bruker 850 MHZ Avance III spectrometer at 25 °C equipped with a cryoprobe. NMR samples contained 2 mM peptide in 10 mM sodium phosphate pH 6.5, and 10% D_2_O, in the absence or presence of 0.03 mM PIN1. Total Correlation Spectroscopy (TOCSY) data for the peptide in the absence of PIN1 was collected with 4096 (TDF2) and 256 data points (TDF1), a spectral width of 10 ppm (8503 Hz) x 10 ppm (8503 Hz), 80 ms mixing time, and 10417 Hz spinlock frequency. ROESY data for the peptide in the presence of PIN1 were acquired with similar data points and spectral width as described for the TOCSY experiment but with different mixing times of 30, 50, 70, 90, 110 ms, and 4310 Hz spinlock frequency. ROESY data for the substrate peptide in the presence and absence of PIN1 were also acquired with 300 ms mixing time. All NMR spectra were processed with Topspin and analyzed with CcpNmr Analysis. The I_tc_/I_tt_ ratios (peak intensity ratio of the conformational exchange *trans*-to-*cis* cross-peak to the *trans* conformation diagonal-peak) for pSer7 and Glu9, which precedes and follows Pro8 respectively, depend on the forward (K_ct_^cat^) and reverse (K_tc_^cat^) rate constants for the two-state *cis*-to-*trans* conformational exchange process. In order to determine K_ct_^cat^ and K_tc_^cat^, tc/tt ratios were fitted to the equation below using KaleidaGraph (Synergy software). I_tc_/I_tt_ = K_tc_^cat^[exp(K_ex_t_m_)-1]/ [K_ct_^cat^exp(K_ex_t_m_) + K_tc_^cat^], where t_m_ is the mixing time, while K_ex_ is the sum of K_ct_^cat^ and K_tc_^cat^

### Protein purification and GST pull-down assay

GST pull-down was performed as previously described [59]. Briefly, for the interaction between GST-PIN1 and Flag-FAAP20 in vitro, GST or GST-PIN1 was expressed using *E*. *coli* BL21 (DE3) expression strain induced with 0.5 mM isopropyl β-D-1-thiogalactopyranoside (IPTG, Sigma-Aldrich) at 30 °C. Cells were lysed in PBS with lysozyme, sonicated, and further incubated with 1% Triton X-100. Cell lysates were recovered by centrifugation at 15,000 rpm at 4 °C for 15 min and incubated with glutathione-sepharose beads (GE Healthcare). After washing, the beads were incubated with in vitro transcribed and translated (IVTT) proteins in NETN150 buffer for 3 h at 4 °C followed by three washes. For IVTT, a total of 250 ng of pcDNA3 plasmids were incubated with 10 μL of TnT T7 Quick Coupled Transcription/Translation Master Mix (Promega) at 30 °C for 70 min to produce proteins. For the purification of recombinant PIN1 for NMR analysis, His-PIN1 was expressed using BL21 (DE3) cells in 2xYT medium with 0.5 mM IPTG at 18 °C overnight. Cells were resuspended and crushed in NiA buffer (20 mM Tris [pH 7.5], 500 mM NaCl, 5% glycerol, and 5 mM imidazole) using the EmulsiFlex-C3 homogenizer (Avestin). The soluble protein was loaded onto Ni-NTA resin, washed, and eluted with NiB buffer (20 mM Tris [pH 7.5], 500 mM NaCl, 5% glycerol, and 1 M imidazole). The eluate was dialyzed against 20 mM Tris [pH 7.5], 500 mM NaCl, 5% glycerol, and stored at -80 °C.

### In vivo ubiquitin assay

In vivo ubiquitin assays were performed under denaturing conditions. MG132-treated cells were resuspended with PBS/1% SDS, snap-frozen in liquid nitrogen, and boiled for 15 min. Cell lysates were diluted 10-fold with PBS and centrifuged at 15,000 rpm for 15 min at 4 °C. Lysate aliquots (4%) were saved for input, and lysates were incubated with HisPur Ni-NTA Resin (Thermo Fisher) in the presence of 10 mM imidazole (Sigma-Aldrich) at 4 °C for 3 h, followed by five washes with PBS/0.1% SDS, 10 mM imidazole. Resins were boiled in 2X Laemmli sample buffer and subjected to SDS-PAGE and Western blotting.

### RT-qPCR

RNA was isolated using TRIzol (Invitrogen). cDNA synthesis was performed using a high-capacity cDNA reverse transcription kit (Applied Biosystems) according to the manufacturer’s protocols. Real-time quantitative PCR was performed using Fast SYBR Green Master Mix (Applied Biosystems) and a StepOnePlus Real-Time PCR system (Applied Biosystems). GAPDH mRNA levels were used as a control for normalization. The following primers were used for cDNA amplification: PP2Ac forward 5′-CAGCTAGTGATGGAGGGATA-3′; PP2Ac reverse 5′-TGGGTCAAACTGCAAGAAA-3′; FBW7 forward 5′-CACTCAAAGT GTGGAATGCAGAGAC-3′; FBW7 reverse 5′-GCATCTCGAGAACCGCTAACAA-3′; GAPDH forward 5′-CAACTACATGGTTTACATGTTC-3′; GAPDH reverse 5′- GCCAGTGGACTCCACGAC-3′.

### CRISPR/Cas9 gene editing

A pair of oligos containing the human *PIN1* sgRNA targeting sequence was designed using crispr.mit.edu. The forward oligo sequence is 5′-CACCGATGCGCTTCTCCCAGCCGGG-3′ and the reverse oligo sequence is 5′-AAACCCCGGCTGGGAGAAGCGCATC-3′. Annealed oligos were cloned into pSpCas9(BB)-2A-Puro (pX459; a gift from Feng Zhang, Addgene plasmid #48139) and transfected into U2OS cells using GeneJuice (Millipore). Control cells were transfected with a pX459 empty vector. Twenty-four hours after transfection, cells were selected with 2 μg/mL puromycin. After recovery from selection, cells were seeded onto 96-well plates, in medium without puromycin, for clonal selection. Selected clones were subsequently analyzed by Western blotting, using anti-PIN1 antibody, to confirm successful knockout.

### Cell survival assay

Cells in 6-well plates were transfected with siRNA oligos and seeded on 96-well plates the next day. Cells were treated with increasing doses of MMC in duplicates at 48 h after transfection, and cell viability was determined using the CellTiter-Glo luminescent cell viability assay (Promega) 4–5 days after continuous drug treatment. Luminescence was measured using a GloMax Navigator microplate luminometer (Promega). Mean values were analyzed for statistical significance using paired Student’s t-test.

### Alkaline comet assay

Single-cell gel electrophoresis for the detection of MMC-induced DNA breaks was performed using the CometAssay kit (4250-050-K, Trevigen) according to the manufacturer’s protocol. Twenty-five μL of a cell suspension at 2 x 10^5^ cells per mL were combined with 225 μL of low-melting agarose (1:10 ratio, vol/vol), and 50 μL were spread on Comet slides (Trevigen). After solidification, the slides were immersed in cold lysing solution at 4 °C for 45 min and placed in freshly prepared alkaline unwinding solution (200 mM NaOH, 1 mM EDTA) for 20 min at RT. Electrophoresis of unwound DNA was performed at 21 V for 30 min. The slides were washed with dH2O for 5 min, dehydrated with 70% ethanol for 5 min, dried, and stained with SYBR Gold (Thermo Fisher). Comet tails were examined using a Nikon Eclipse E600 fluorescence microscope and analyzed by OpenComet [61]. Per group, up to 300 individual nuclei were evaluated. The olive tail moment was calculated as a measure of DNA damage and presented as the product of the DNA % (tail intensity) and the distance between the intensity-weighted centroids of a head and a tail (DNA migration). Difference between mean values was tested for statistical significance using two-tailed unpaired Student’s t-test.

### Flow cytometry

For quantification of γH2AX-positive cells, cell pellets were pre-extracted with PBS/0.5% Triton-X for 5 min, fixed with 4% paraformaldehyde for 15 min, and incubated with γH2AX Alexa Fluor 488 (1:100; CR55T33, Thermo Fisher) in Foxp3/transcription factor staining buffer (Thermo Fisher) for 1 h. Cells were washed once and suspended in 500 μL 7-AAD viability staining solution (Thermo Fisher) supplemented with 200 μg/mL PureLink RNase A, stained for 30 min at 37 °C, and analyzed by Attune NxT acoustic focusing cytomether and Attune NxT software v2.7 (Thermo Fisher).

### Statistical analysis

Student’s t-test was used to assess the statistical significance of our results, using Prism (GraphPad).

## Supporting information

S1 Fig Characterization of the FAAP20 WLR by mass spectrometry. **(A)** Silver staining and Western blotting (WB) of Flag-FAAP20 WT and WLR mutant purified from 293T cells by anti-Flag immunoprecipitation (IP) and elution with Flag peptide. The Flag-FAAP20 WLR mutant exhibits an additional slower migrating isoform (U) alongside the isoform observed in the WT (L). **(B)** Mass spectrometry (MS) analysis of the purified Flag-FAAP20 WLR isoforms. Both upper and lower bands from Flag-FAAP20 WLR were excised and analyzed by MS. The pSer48 residue shaded in blue is specifically present in the upper isoform of Flag-FAAP20 WLR. **(C)** Annotated MS/MS spectrum for 39–69 peptide of the FAAP20 WLR mutant phosphorylated at Ser48. The sequence of the peptide is displayed horizontally and in the right-hand vertical panel. The phosphorylated serine is indicated by the lower case ‘s’ in red at residue 48. The y and b ions annotated in the figure are indicated by the encircled ion number in the right-hand vertical sequence. Evidence that phosphorylation occurs at Ser48 is supported by the strong y_22_^2+^ spectral peak (*) and the identified y ion series surrounding this ion. **(D)** (Top) 293T cells transiently transfected with indicated plasmids were analyzed by WB. Immunoblots were quantitated by ImageJ, and the U/L ratio was derived from the average of two independent experiments. (Bottom) U2OS cells were serially transfected with PIN1 siRNA (vs. control) and Flag-FAAP20 WLR, and lysates were analyzed by WB.
(TIF)

S2 Fig Interaction between PIN1 and FAAP20. **(A)** Lysates from 293T cells were incubated with glutathione beads bound with GST or GST-PIN1 and the levels of precipitated endogenous FAAP20 was analyzed by WB. **(B)** In vitro transcribed and translated (IVTT) FAAP20 WT, WLR point or deletion mutants were immunoprecipitated by anti-Flag agarose and analyzed by WB.
(TIF)

S3 Fig Analysis of the pFAAP20 peptide isomerization rate catalyzed by PIN1. **(A)** Shown are the ratios of cross-peak and diagonal-peak intensities (I_tc_/I_tt_) for the *trans*-to-*cis* conformational change of pSer7 and Glu9 over increasing mixing time as well as its isomerization rate (K_tc_^cat^). For the determination of K_ct_^cat^ and K_tc_^cat^, tc/tt ratios were fitted to the equation given in the Materials and Methods. **(B)** Mean values of the *cis*-to-*trans* isomerization rate (K_tc_^cat^ and K_ct_^cat^) of pSer7 and Glu9 are indicated. The *cis*-to-*trans* conformational exchange rate is enhanced 8.72-fold (K_ct_^cat^ / K_tc_^cat^ = 8.72).
(TIF)

S4 Fig PIN1 knockout promotes FAAP20 degradation. **(A)** U2OS WT or *PIN1*^*-/-*^ #1 clones expressing Flag-FAAP20 were treated with 50 μg/mL CHX for the indicated times and degradation of Flag-FAAP20 was analyzed by WB. **(B)** Quantification of Flag-FAAP20 levels of Fig 4E *PIN1*^*-/-*^ #6 from two independent experiments. * *p* <0.01, unpaired two-tailed t-test. **(C)** Quantification of Flag-FAAP20 levels of Fig 4H from two independent experiments. * *p* <0.05, unpaired two-tailed t-test. **(D)** U2OS WT or *PIN1*^*-/-*^ #6 clones cells transfected with the indicated plasmids were treated with 10 μM MG132 for 6 h, lysed under denaturing conditions, and incubated with Ni-NTA agarose to capture polyubiquitinated Flag-FAAP20.
(TIF)

S5 Fig Confirmation of antibody and siRNA. **(A)** 293T cells expressing Flag-FAAP20 wild-type, S113A/S117A, or S48A mutant were treated with 10 μM MG132 for 4 h and pS113 levels were analyzed by WB. **(B)** U2OS cells serially transfected with siRNA PP2Ac-1 and -2 (vs. control) and HA-PP2Ac-encoding plasmid were analyzed by anti-HA WB to confirm the specific targeting of siRNA PP2Ac to PP2Ac cDNA.
(TIF)

S6 Fig The FAAP20-GSKβ interaction and confirmation of knockdown. **(A)** 293T cells were transfected with indicated plasmids, and the amount of HA-GSKβ pulled-down by Flag-FAAP20 was analyzed by anti-Flag IP and WB. **(B)** Confirmation of *FBW7* knockdown by RT-qPCR. mRNA expression was normalized by GAPDH mRNA (mean ± SD; n = 2 independent experiments of duplicated samples), * *P* <0.001, Student’s t-test.
(TIF)

S7 Fig Characterization of the PIN1-depleted cells. **(A)** U2OS cells serially transfected with siRNA PIN1 (vs. control) and Flag-FAAP20 CPD (S113A & S117A) (vs. EV) were treated with 100 μg/mL CHX for the indicated times, and cell lysates were analyzed by WB. A short-lived protein MCL-1 serves as a control for CHX treatment. Endogenous FANCA levels were quantified using ImageJ from two independent experiments. **(B)** U2OS cells transfected with indicated siRNA oligos were analyzed by WB. **(C)** (Left) WB analysis of U2OS cells depleted of FAAP20 and reconstituted with siRNA-resistant pMSCV-Flag-HA (F/H)-tagged FAAP20 WT, ΔWLR (a.a.40-45 deletion), or CPD (S113A & S117A). (Right) cellular viability of U2OS cells reconstituted as above. Data shown are mean ± SEM from three independent experiments. * *P* <0.05, WT vs. WLR reconstitution, paired two-tailed Student’s t-test. **(D)** The viability of MDA-MB-231 cells treated with indicated concentration of ATRA for 72 h was determined by luminescence-based quantification of cellular ATP levels. Mean ± SD; n = 3 independent experiments, n.s. not significant, Student’s t-test. **(E)** 293T cells transiently transfected with indicated Flag-FAAP20 plasmids were subjected to Flag IP, and co-immunoprecipitated endogenous FANCA was analyzed by WB.
(TIF)
