# Supplementary figures and images for: Prolyl isomerization of FAAP20 catalyzed by PIN1 regulates the Fanconi anemia pathway
Source: PLoS Genet. 2019 Feb 21;15(2):e1007983. doi: 10.1371/journal.pgen.1007983 (PMC6400411; doi:10.1371/journal.pgen.1007983)

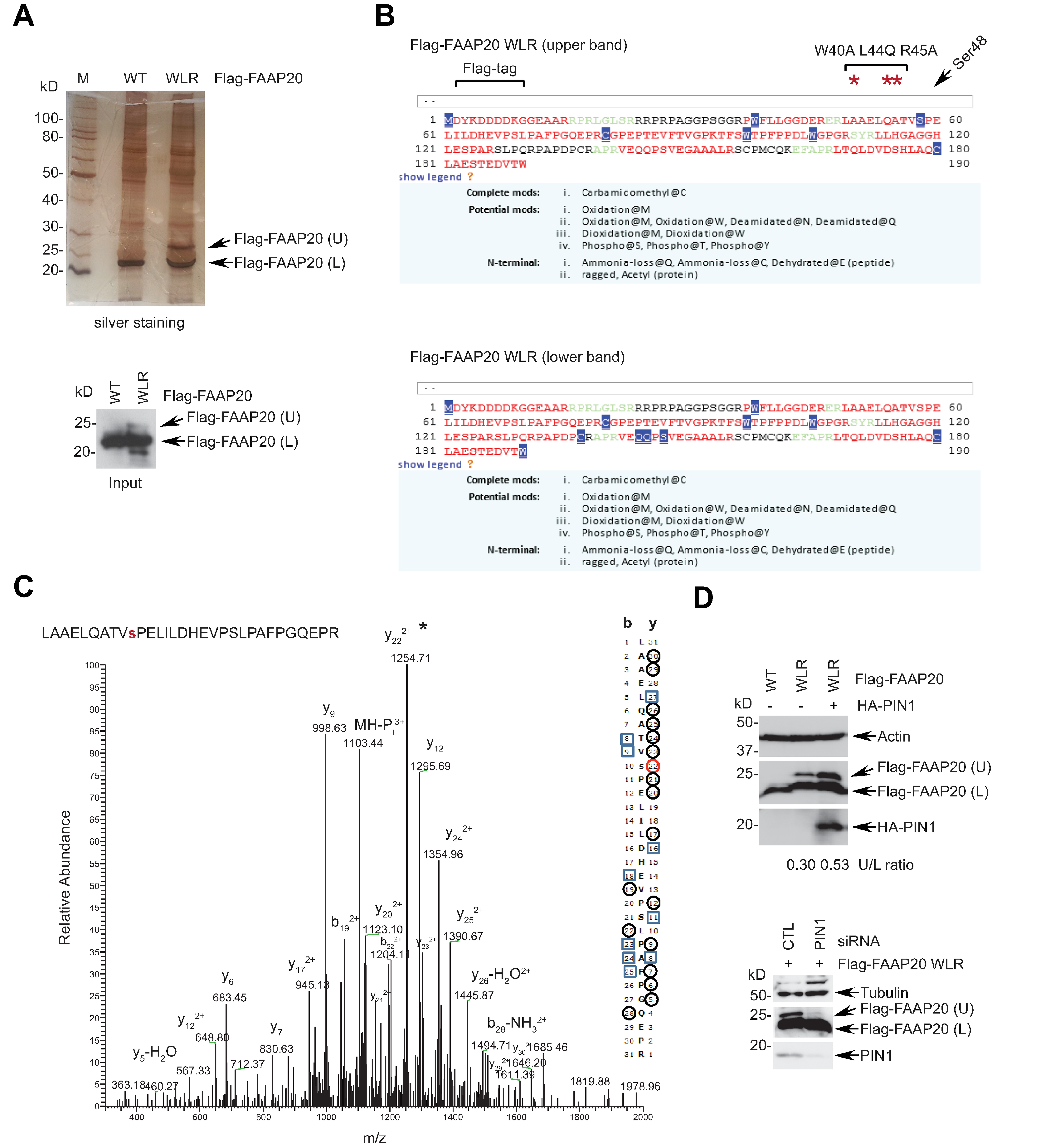

Supplement: S1 Fig — (A) Silver staining and Western blotting (WB) of Flag-FAAP20 WT and WLR mutant purified from 293T cells by anti-Flag immunoprecipitation (IP) and elution with Flag peptide. The Flag-FAAP20 WLR mutant exhibits an additional slower migrating isoform (U) alongside the isoform observed in the WT (L). (B) Mass spectrometry (MS) analysis of the purified Flag-FAAP20 WLR isoforms. Both upper and lower bands from Flag-FAAP20 WLR were excised and analyzed by MS. The pSer48 residue shaded in blue is specifically present in the upper isoform of Flag-FAAP20 WLR. (C) Annotated MS/MS spectrum for 39–69 peptide of the FAAP20 WLR mutant phosphorylated at Ser48. The sequence of the peptide is displayed horizontally and in the right-hand vertical panel. The phosphorylated serine is indicated by the lower case ‘s’ in red at residue 48. The y and b ions annotated in the figure are indicated by the encircled ion number in the right-hand vertical sequence. Evidence that phosphorylation occurs at Ser48 is supported by the strong y222+ spectral peak (*) and the identified y ion series surrounding this ion. (D) (Top) 293T cells transiently transfected with indicated plasmids were analyzed by WB. Immunoblots were quantitated by ImageJ, and the U/L ratio was derived from the average of two independent experiments. (Bottom) U2OS cells were serially transfected with PIN1 siRNA (vs. control) and Flag-FAAP20 WLR, and lysates were analyzed by WB. (TIF) [file pgen.1007983.s001.tif]

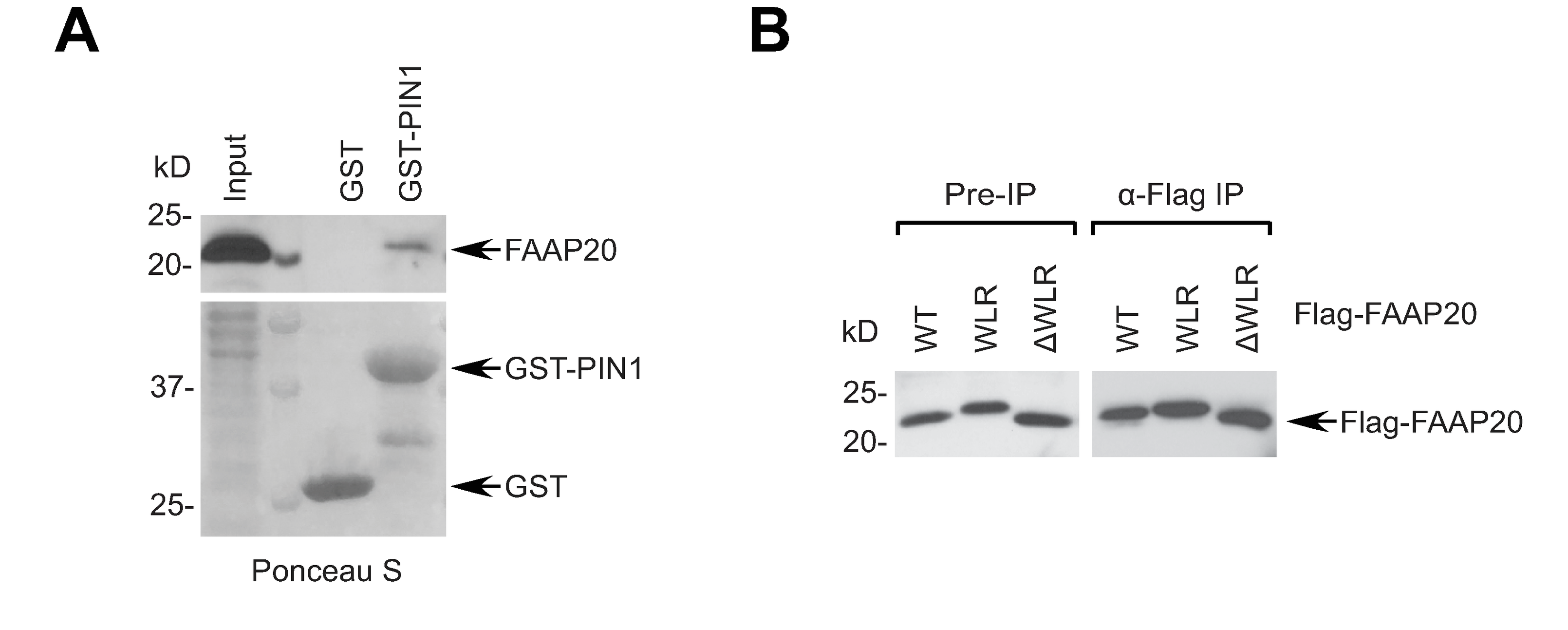

Supplement: S2 Fig — (A) Lysates from 293T cells were incubated with glutathione beads bound with GST or GST-PIN1 and the levels of precipitated endogenous FAAP20 was analyzed by WB. (B) In vitro transcribed and translated (IVTT) FAAP20 WT, WLR point or deletion mutants were immunoprecipitated by anti-Flag agarose and analyzed by WB. (TIF) [file pgen.1007983.s002.tif]

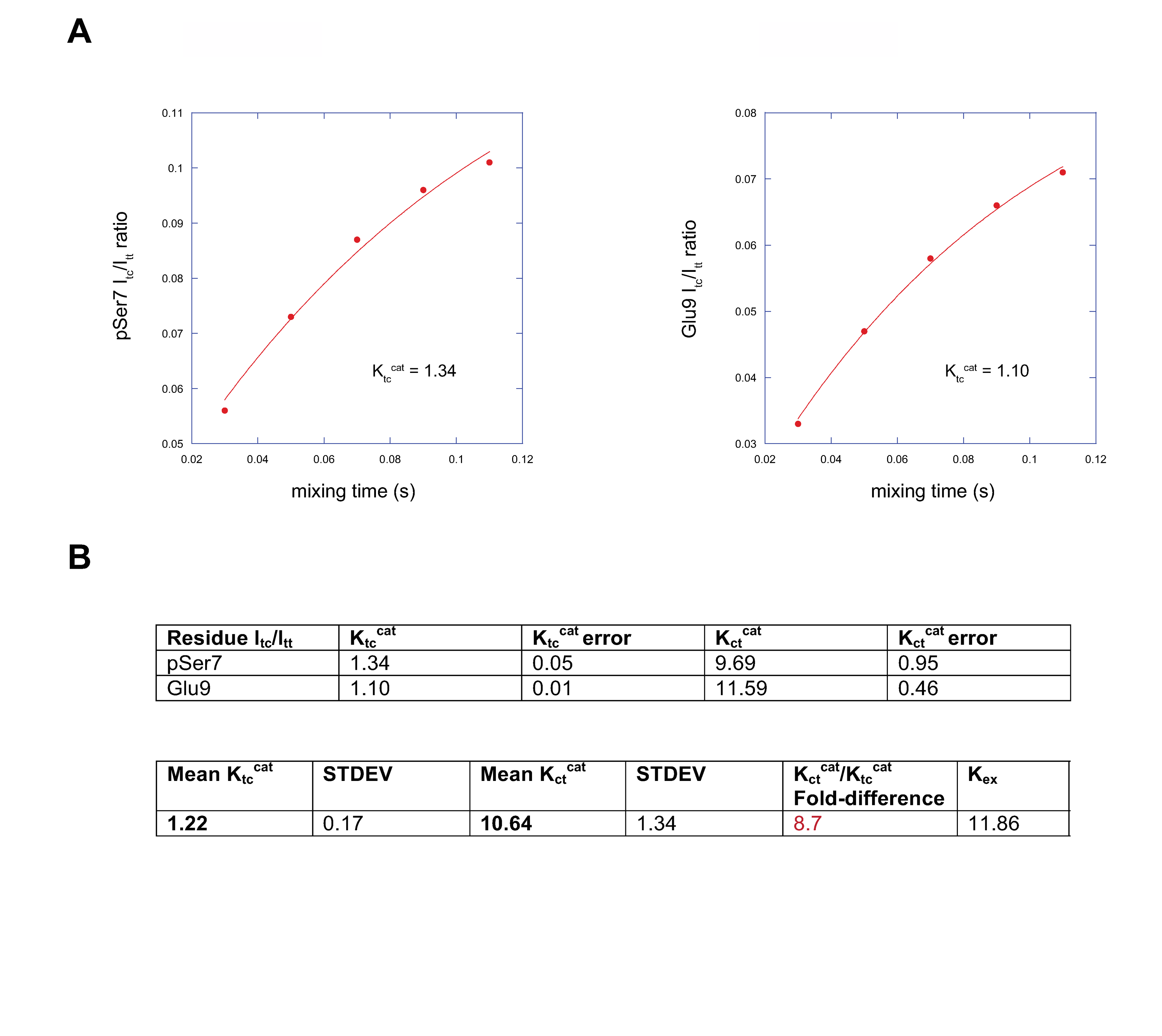

Supplement: S3 Fig — (A) Shown are the ratios of cross-peak and diagonal-peak intensities (Itc/Itt) for the trans-to-cis conformational change of pSer7 and Glu9 over increasing mixing time as well as its isomerization rate (Ktccat). For the determination of Kctcat and Ktccat, tc/tt ratios were fitted to the equation given in the Materials and Methods. (B) Mean values of the cis-to-trans isomerization rate (Ktccat and Kctcat) of pSer7 and Glu9 are indicated. The cis-to-trans conformational exchange rate is enhanced 8.72-fold (Kctcat / Ktccat = 8.72). (TIF) [file pgen.1007983.s003.tif]

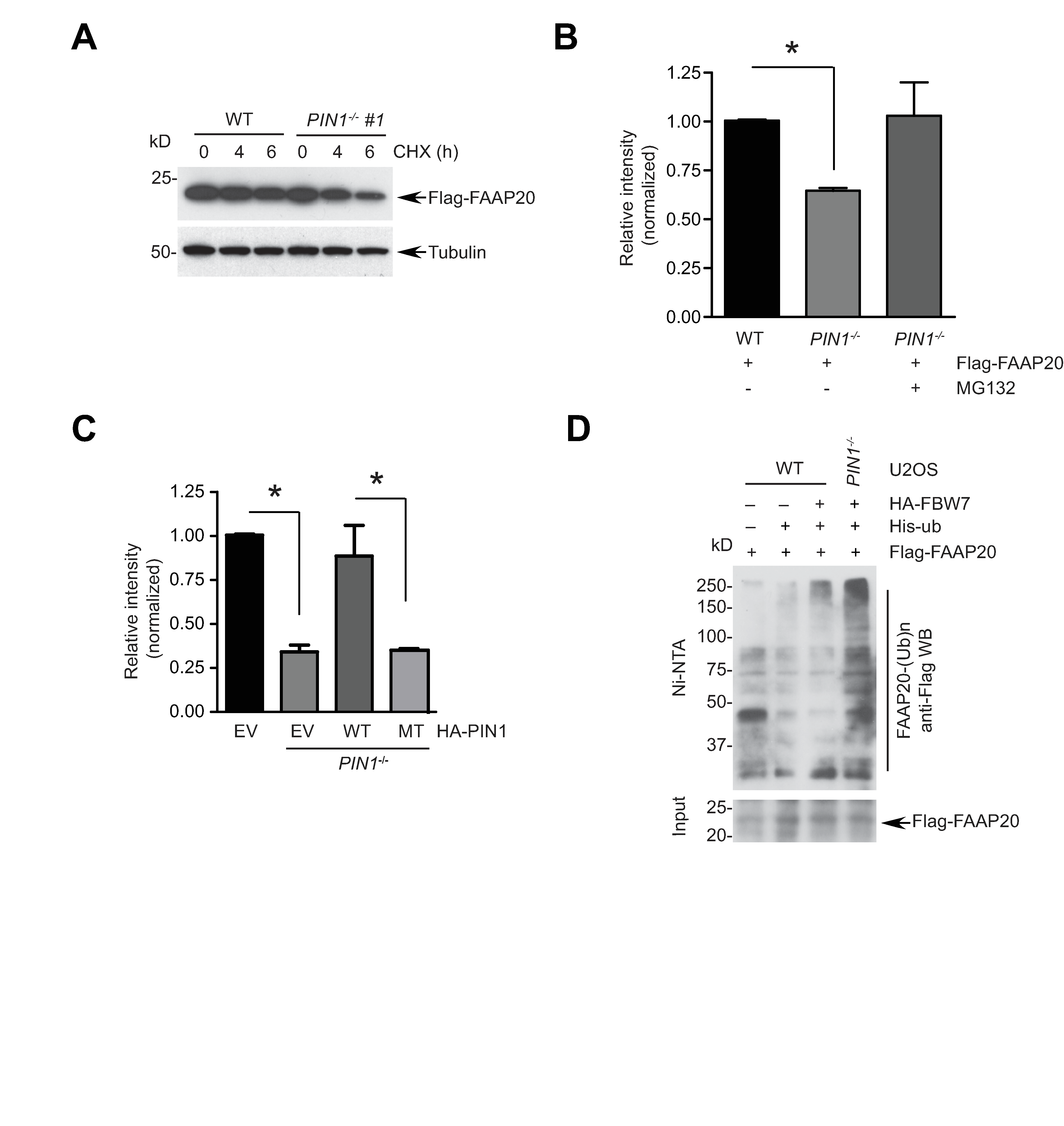

Supplement: S4 Fig — (A) U2OS WT or PIN1-/- #1 clones expressing Flag-FAAP20 were treated with 50 μg/mL CHX for the indicated times and degradation of Flag-FAAP20 was analyzed by WB. (B) Quantification of Flag-FAAP20 levels of Fig 4E PIN1-/- #6 from two independent experiments. * p <0.01, unpaired two-tailed t-test. (C) Quantification of Flag-FAAP20 levels of Fig 4H from two independent experiments. * p <0.05, unpaired two-tailed t-test. (D) U2OS WT or PIN1-/- #6 clones cells transfected with the indicated plasmids were treated with 10 μM MG132 for 6 h, lysed under denaturing conditions, and incubated with Ni-NTA agarose to capture polyubiquitinated Flag-FAAP20. (TIF) [file pgen.1007983.s004.tif]

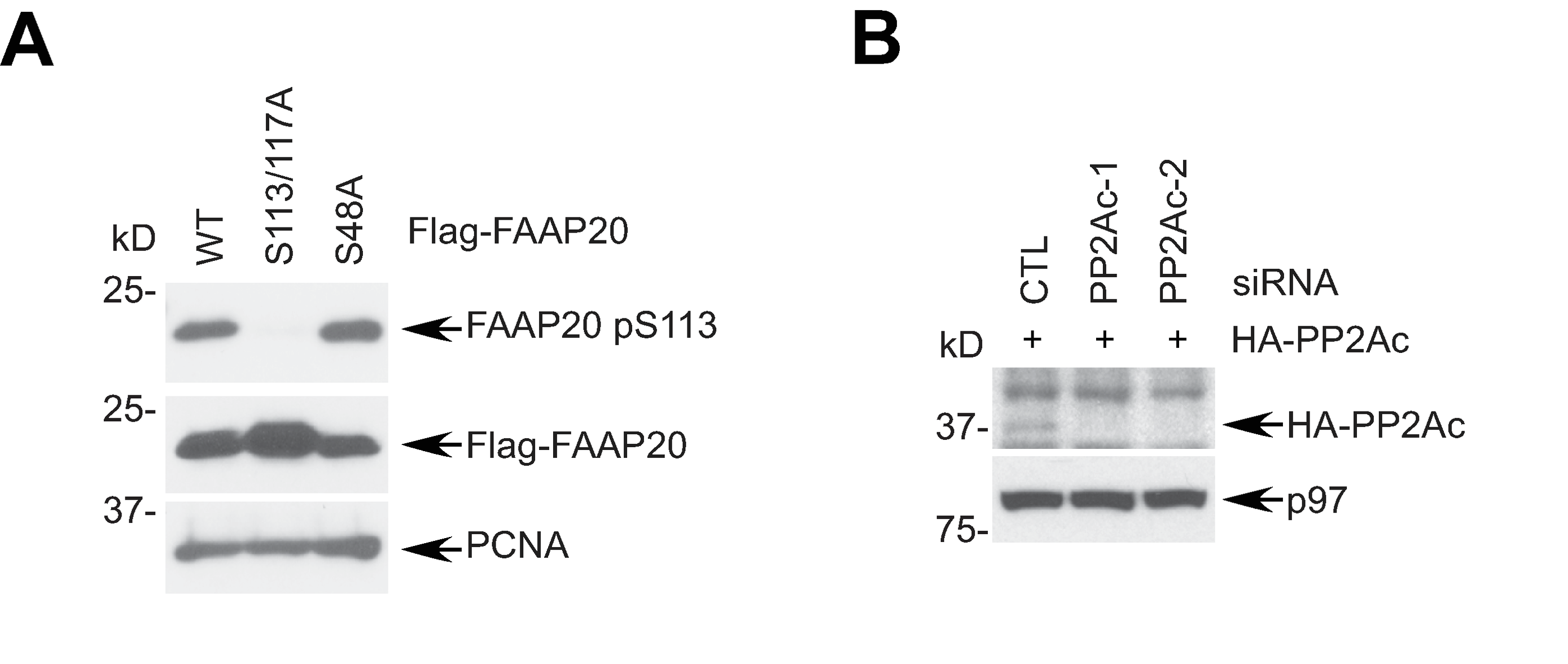

Supplement: S5 Fig — (A) 293T cells expressing Flag-FAAP20 wild-type, S113A/S117A, or S48A mutant were treated with 10 μM MG132 for 4 h and pS113 levels were analyzed by WB. (B) U2OS cells serially transfected with siRNA PP2Ac-1 and -2 (vs. control) and HA-PP2Ac-encoding plasmid were analyzed by anti-HA WB to confirm the specific targeting of siRNA PP2Ac to PP2Ac cDNA. (TIF) [file pgen.1007983.s005.tif]

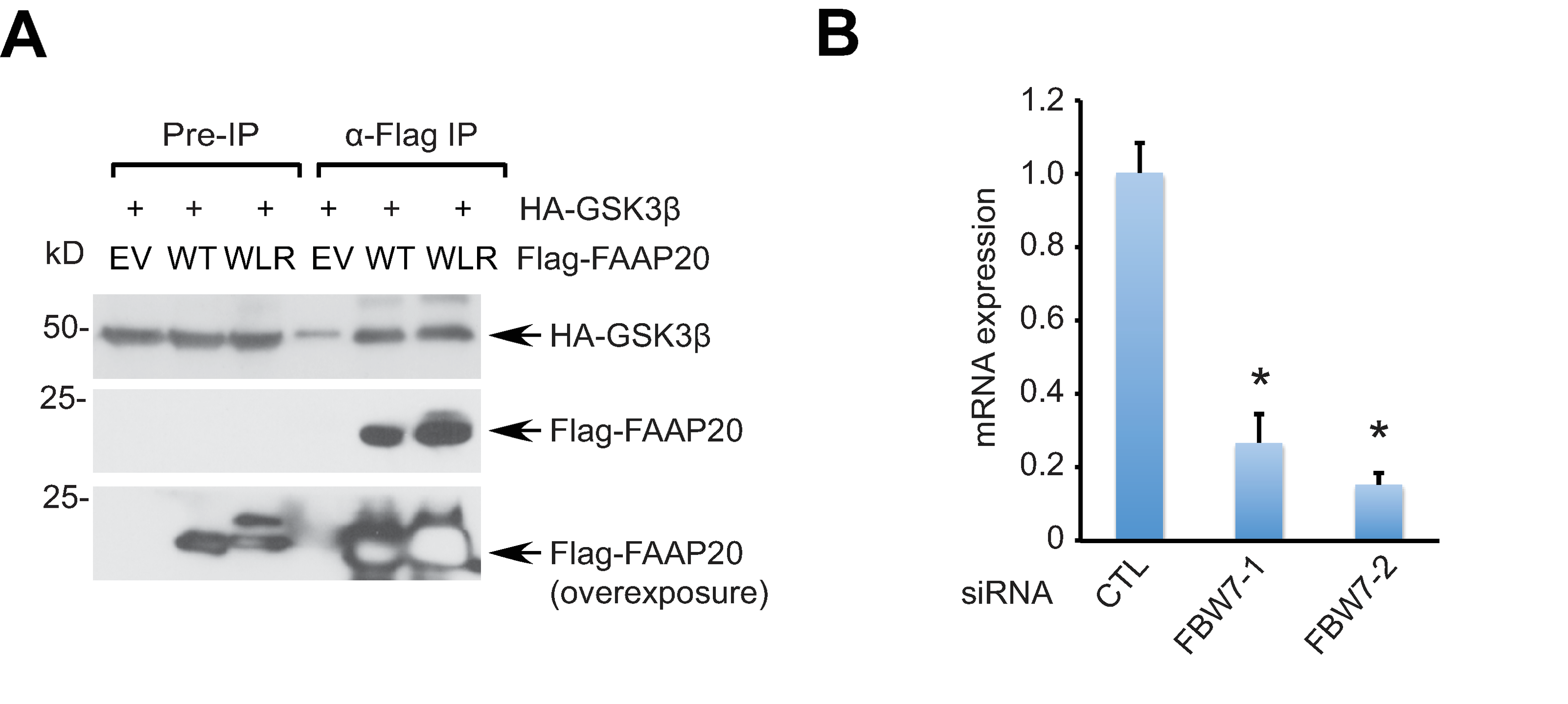

Supplement: S6 Fig — (A) 293T cells were transfected with indicated plasmids, and the amount of HA-GSKβ pulled-down by Flag-FAAP20 was analyzed by anti-Flag IP and WB. (B) Confirmation of FBW7 knockdown by RT-qPCR. mRNA expression was normalized by GAPDH mRNA (mean ± SD; n = 2 independent experiments of duplicated samples), * P <0.001, Student’s t-test. (TIF) [file pgen.1007983.s006.tif]

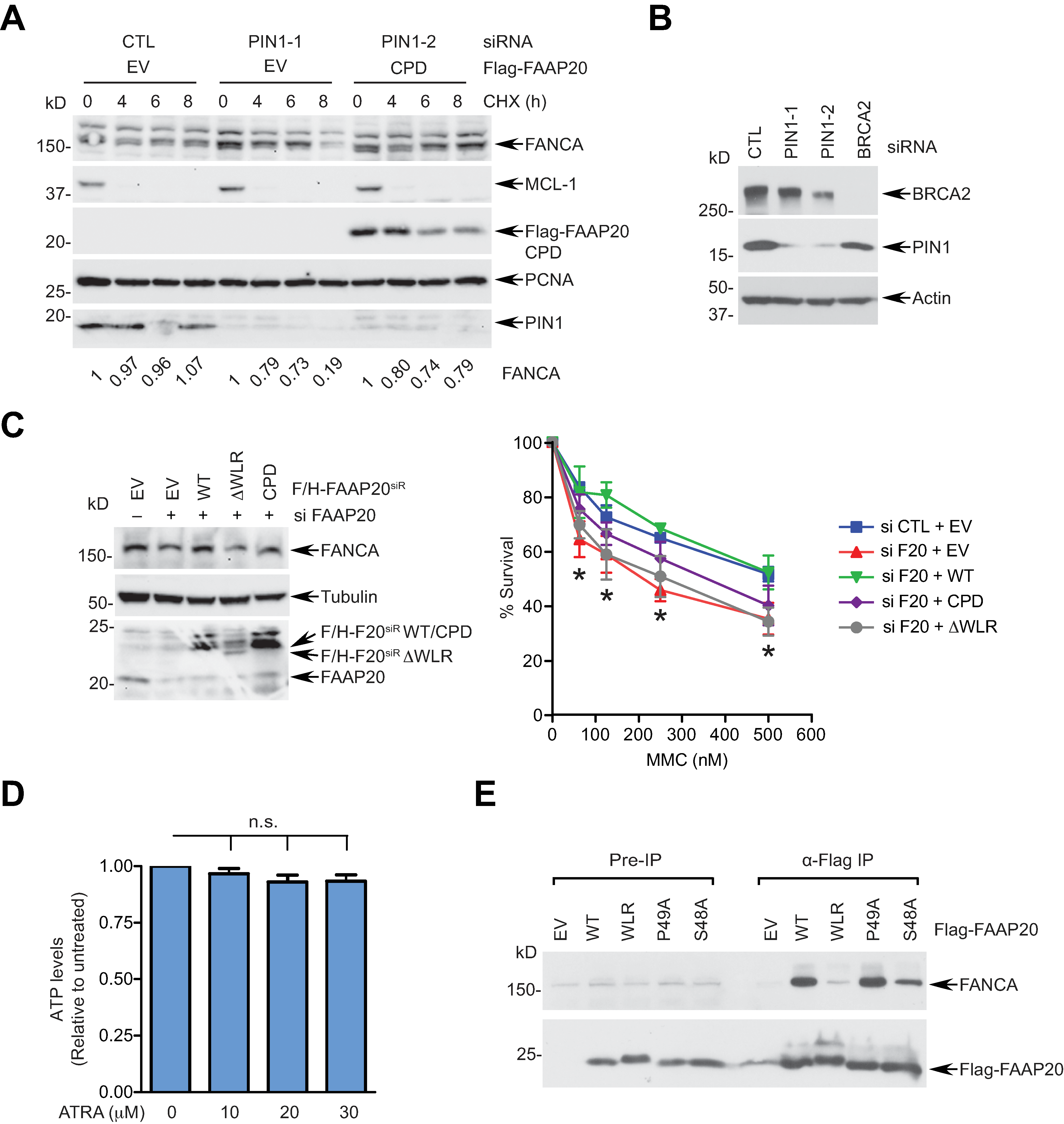

Supplement: S7 Fig — (A) U2OS cells serially transfected with siRNA PIN1 (vs. control) and Flag-FAAP20 CPD (S113A & S117A) (vs. EV) were treated with 100 μg/mL CHX for the indicated times, and cell lysates were analyzed by WB. A short-lived protein MCL-1 serves as a control for CHX treatment. Endogenous FANCA levels were quantified using ImageJ from two independent experiments. (B) U2OS cells transfected with indicated siRNA oligos were analyzed by WB. (C) (Left) WB analysis of U2OS cells depleted of FAAP20 and reconstituted with siRNA-resistant pMSCV-Flag-HA (F/H)-tagged FAAP20 WT, ΔWLR (a.a.40-45 deletion), or CPD (S113A & S117A). (Right) cellular viability of U2OS cells reconstituted as above. Data shown are mean ± SEM from three independent experiments. * P <0.05, WT vs. WLR reconstitution, paired two-tailed Student’s t-test. (D) The viability of MDA-MB-231 cells treated with indicated concentration of ATRA for 72 h was determined by luminescence-based quantification of cellular ATP levels. Mean ± SD; n = 3 independent experiments, n.s. not significant, Student’s t-test. (E) 293T cells transiently transfected with indicated Flag-FAAP20 plasmids were subjected to Flag IP, and co-immunoprecipitated endogenous FANCA was analyzed by WB. (TIF) [file pgen.1007983.s007.tif]
